# Assembly and disassembly of *Aspergillus fumigatus* conidial rodlets

**DOI:** 10.1016/j.tcsw.2019.100023

**Published:** 2019-03-06

**Authors:** Isabel Valsecchi, Jennifer I. Lai, Emmanuel Stephen-Victor, Ariane Pillé, Audrey Beaussart, Victor Lo, Chi L.L. Pham, Vishukumar Aimanianda, Ann H. Kwan, Magalie Duchateau, Quentin Giai Gianetto, Mariette Matondo, Melanie Lehoux, Donald C. Sheppard, Yves F. Dufrene, Jagadeesh Bayry, J. Iñaki Guijarro, Margaret Sunde, Jean-Paul Latgé

**Affiliations:** a Unité des Aspergillus, Institut Pasteur, Paris, France; b School of Medical Sciences and Sydney Nano, The University of Sydney, NSW 2006, Australia; c Institut National de la Santé et de la Recherche Médicale, Centre de Recherche des Cordeliers, Sorbonne Université, Paris, France; d Biological NMR Technological Platform, Institut Pasteur, CNRS UMR 3528, Paris, France; e Institute of Life Sciences, Université Catholique de Louvain, Croix du Sud, 4-5, bte L7.07.06, B-1348 Louvain-la-Neuve, Belgium; f Walloon Excellence in Life Sciences and Biotechnology, Belgium; g School of Life and Environmental Sciences and Sydney Nano, The University of Sydney, NSW 2006, Australia; h Pasteur Proteomics Platform, Mass Spectrometry for Biology Unit, Institut Pasteur, CNRS USR 2000, Paris, France; i Bioinformatics and Biostatistics Hub, C3BI, CNRS USR 3756, Institut Pasteur, Paris, France; j Departments of Medicine, Microbiology and Immunology, McGill University, Montréal, QC, Canada

**Keywords:** *Aspergillus fumigatus*, Hydrophobins, Rodlets, RodA, Amyloids

## Abstract

The rodlet structure present on the *Aspergillus fumigatus* conidial surface hides conidia from immune recognition. In spite of the essential biological role of the rodlets, the molecular basis for their self-assembly and disaggregation is not known. Analysis of the soluble forms of conidia-extracted and recombinant RodA by NMR spectroscopy has indicated the importance of disulfide bonds and identified two dynamic regions as likely candidates for conformational change and intermolecular interactions during conversion of RodA into the amyloid rodlet structure. Point mutations introduced into the *RODA* sequence confirmed that (1) mutation of a single cysteine was sufficient to block rodlet formation on the conidial surface and (2) both presumed amyloidogenic regions were needed for proper rodlet assembly. Mutations in the two putative amyloidogenic regions retarded and disturbed, but did not completely inhibit, the formation of the rodlets *in vitro* and on the conidial surface. Even in a disturbed form, the presence of rodlets on the surface of the conidia was sufficient to immunosilence the conidium. However, in contrast to the parental conidia, long exposure of mutant conidia lacking disulfide bridges within RodA or expressing RodA carrying the double (I115S/I146G) mutation activated dendritic cells with the subsequent secretion of proinflammatory cytokines. The immune reactivity of the RodA mutant conidia was not due to a modification in the RodA structure, but to the exposure of different pathogen-associated molecular patterns on the surface as a result of the modification of the rodlet surface layer. The full degradation of the rodlet layer, which occurs during early germination, is due to a complex array of cell wall bound proteases. As reported earlier, this loss of the rodlet layer lead to a strong anti-*fumigatus* host immune response in mouse lungs.

## 1 Introduction

Aerial conidia of Ascomycetes and Basidiomycetes are coated with a layer of hydrophobin proteins organized in fibrillar structures known as rodlets. The rodlet layer is amphipathic, and the outward facing hydrophobic surface renders the conidial surface resistant to wetting, thus facilitating effective dispersal of conidia in the air (Gebbink et al., 2005, Sunde et al., 2008). Fungal hydrophobins are small secreted proteins with eight cysteine residues ordered in a distinct way and with characteristic hydropathy patterns, which define two well-characterized classes I and II and a third intermediate class named III (Gandier et al., 2017, Kershaw and Talbot, 1998, Littlejohn et al., 2012, Pedersen et al., 2011, Wessels, 1994). Class I hydrophobin assemblies are generally water- and detergent-insoluble, have an amyloid structure and form the patterned rodlet layer observed in conidia (Butko et al., 2001, de Vocht et al., 2000, Mackay et al., 2001). Class II hydrophobin sequences are generally shorter than class I hydrophobin sequences and are soluble in aqueous ethanol mixtures and SDS. The intermediate class III has the same cysteine pattern but lacks a consistent hydropathy profile characteristic of either class I or class II hydrophobins (Jensen et al., 2010, Littlejohn et al., 2012).

In addition to the role of the rodlet layer in fungal life during conidiogenesis and conidial dispersal in the air, it has been shown that rodlets have a role in fungal–host interactions and favor the pathogenic behaviour of fungal pathogens in mammals, plant and insect hosts (Aimanianda et al., 2009, Talbot et al., 1996, Zhang et al., 2011) or play a positive role during plant symbiosis (Whiteford and Spanu, 2002). Moreover, due to their specific physicochemical properties, hydrophobins have potential for numerous biotechnological applications (Piscitelli et al., 2017, Wösten and Scholtmeijer, 2015, Zhao et al., 2009). Their coating properties could be used to functionalize metals and plastic or carbon nanotubes in order to prevent the formation of bacterial biofilms or to immobilize enzymes on surface (Tanaka et al., 2017) or to stabilize pharmaceutical emulsions and facilitate drug delivery (Artini et al., 2017).

Our current study is focused on the RodA hydrophobin of *A. fumigatus*, the major airborne opportunistic fungal pathogen of humans. This hydrophobin forms rodlets on the surface of *A. fumigatus* conidia (Paris et al., 2003, Thau et al., 1994). This surface rodlet coat masks the conidium from host detection and its removal from the conidial surface initiates the antifungal host immune response (Aimanianda et al., 2009)*.* Despite their prominent morphological and biological role, the molecular basis for rodlet self-assembly and disaggregation in this fungal species has not yet been understood. By analysing the structure of the soluble form of RodA and by creating point mutations in the *RODA* gene, we report here on the mechanism that drives RodA to assemble into highly ordered structures anchored to the conidial surface. Moreover, we have also investigated the biochemical events responsible for the lysis of the rodlet layer, a key step in the recognition and mounting of an immune response to this airborne pathogen by host cells.

## 2 Results

### 2.1 Solution structure and dynamics of RodA

**Fig. 1:**
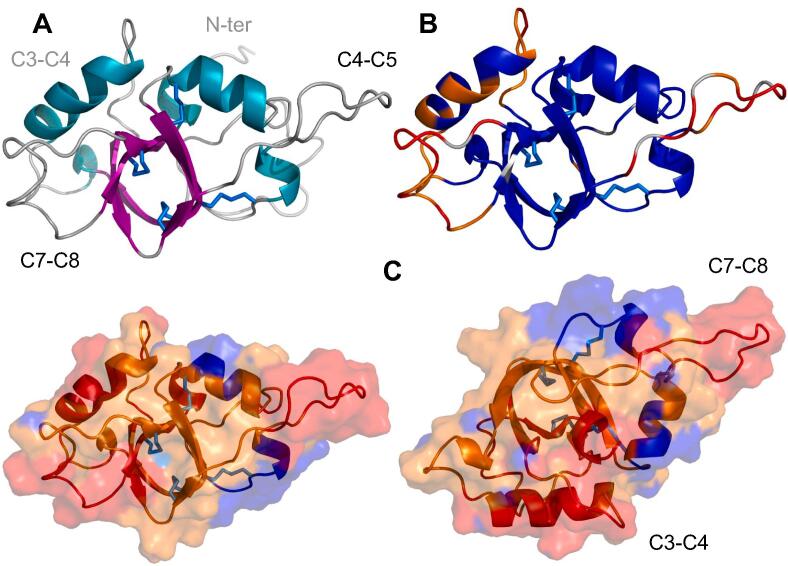
Structure (A), internal dynamics (B) and hydrophobicity (C) of monomeric RodA (best energy conformer). A: Strands are shown in magenta, helices in cyan and disulfide bridges in marine blue. The inter cysteine regions on the front (grey) and back (light grey) as well as the N-terminal region are indicated. Cysteines are numbered by their order in the sequence throughout this paper. B: Backbone amide 1H-15N heteronuclear nOe values are shown on the cartoon representation of the structure: highly flexible (nOe < 0.45), flexible (0.45 ≤ nOe ≤ 0.65), rigid (nOe > 0.65) and missing-value backbone residues are shown in red, orange, blue and grey, respectively. The structure is shown in a different orientation on the right. C: The hydropathy profile calculated with the Eisenberg hydrophobicity scale (http://web.expasy.org/protscale) is color coded on the surface and cartoon representations of the structure: blue = hydrophilic (Φ ≤ −0.3), orange (−0.3 < Φ < 0.3), red = hydrophobic (Φ ≥ 0.3). The N-terminal disordered region (residues 18–38) is not displayed in B and C. (For interpretation of the references to color in this figure legend, the reader is referred to the web version of this article).

The structure of RodA was studied by NMR spectroscopy. Comparison of the ^1^H-^15^N HSQC spectra of recombinant RodA (rRodA) and the HPLC purified hydrofluoric acid-extracted RodA from conidia indicated that both proteins have the same structure, the same cysteine redox state and potential disulfide pairing (Supplementary Material Fig. S1). Additionally, the native protein did not contain any post-translational modification such as an O- or N-glycosylation. This was confirmed by mass spectrometry (MS) of proteolysis fragments of the full-length conidia-extracted protein before or after chemical treatment with the N- and O-deglycosylating agent trifluoromethanesulfonic acid (data not shown). We thus performed further work using the recombinant protein expressed in *E. coli* that could be purified in greater amounts than the native conidial RodA. Analysis of the cysteine redox state as proposed by Sharma Rajarathnam (Sharma and Rajarathnam, 2000) indicated that the Cys residues of rRodA were oxidized and hence, involved in disulfide bridges. Distance (nOe) data and topology of rRodA allowed us to unambiguously establish the disulfide pairing of rRodA, which corresponded to the one observed in all other studied hydrophobins (C1-C6: 56-133; C2-C5: 64-127; C3-C4: 65-105; C7-C8: 134-152) (Fig. 1). Cysteines are numbered according to their order of appearance in the sequence from C1 to C8 or by their sequence number throughout the manuscript.

The structure of RodA (Fig. 1 and Supplementary Table S1) is organized around the four-disulfide bonds. It consists of a central β-barrel composed of two curved antiparallel β-sheets (sheet S1: strands 60–65, 104–107, 130–135 and 150–154; sheet S2: strands 65–70, 99–104 and 150–154), two relatively long α-helices (helix H1: 49–56; H2: 79–90), one short α-helix (H4: 123–126) and two short 3_10_- (H3: 96–98; H5: 155–157) helices. Two long and kinked β-strands (60–70 and 99–107) participate in both β-sheets of the β-barrel. Two S-S bonds link together strands of the β-barrel and the other two connect the external face of the β-barrel with the regions of helices H1 and H4. The long α-helix H2 in the C3-C4 region packs against the β-barrel. The structures showed good convergence within the regions with secondary structure, but were more variable for residues 19–39, 109–121 and 137–149 located in the N-terminal, C4-C5 (106–132) and C7-C8 (135–151) inter-cysteine regions, respectively (Fig. S2).

The internal motions of rRodA on the nanosecond-picosecond time scale were analyzed using the backbone amide ^1^H-^15^N nOes (Fig. 1B and Supplementary Fig. S3). Low nOe values are indicative of high amplitude internal motions while high nOe values reflect rigidity of the backbone at the fast timescale (ns-ps). The nOe data showed that the secondary structure elements of RodA in the vicinity of disulfide bridges, namely the central β-barrel and α-helices H1 and H4 that are tethered to the β-barrel by S-S bonds are rigid with nOe values higher than 0.71. Helix H2, located in the region between cysteine residues C3 and C4, showed somewhat lower ^1^H-^15^N nOe values (0.56 to 0.85) suggestive of low amplitude motions. In contrast, the N-terminal region (19–39) showed negative or very low nOes that indicated that this region was disordered. Residues 109–121 (C4-C5 region), 137–149 (C7-C8 region) and to a lesser extent the flanking regions (74–79 and 90–96) of α-helices H2 and H3 in the C3-C4 region also display low nOe values.

The charge distribution and hydrophobicity of the surface of hydrophobins is important for the recruitment and self-assembly of hydrophobins at a hydrophobic/hydrophilic interface such as the air/water interface (Fig. 1C). The surface of RodA contains a hydrophobic region between C3 and C4 and two highly hydrophobic patches (114–120 and 136–148) in the inter-cysteine regions C4-C5 and C7-C8. A relatively hydrophobic belt joins the hydrophobic and flexible C4-C5 and C7-C8 loops. The belt is lined by charged residues with two pairs of charged residues (E54-K50; K107-D109) that establish salt bridges and compensate their charges. An opposite face of the molecule shows many charged residues with two clusters of negative residues (D73, D76, D78 and E79 in the C3-C4 region and D45 and D46 close to the disordered N-terminal tail) and isolated positive residues (K67, K128 and K55 and K87). Hence, in solution, monomeric RodA shows an amphiphilic character with a hydrophobic face with hydrophobic residues and compensated charges and a more hydrophilic surface with clusters of net charges on the opposite face.

### 2.2 Molecular identification of the amyloidogenic regions within RodA protein and the amino acids essential for the formation of the rodlets *in vitro*

**Fig. 2:**
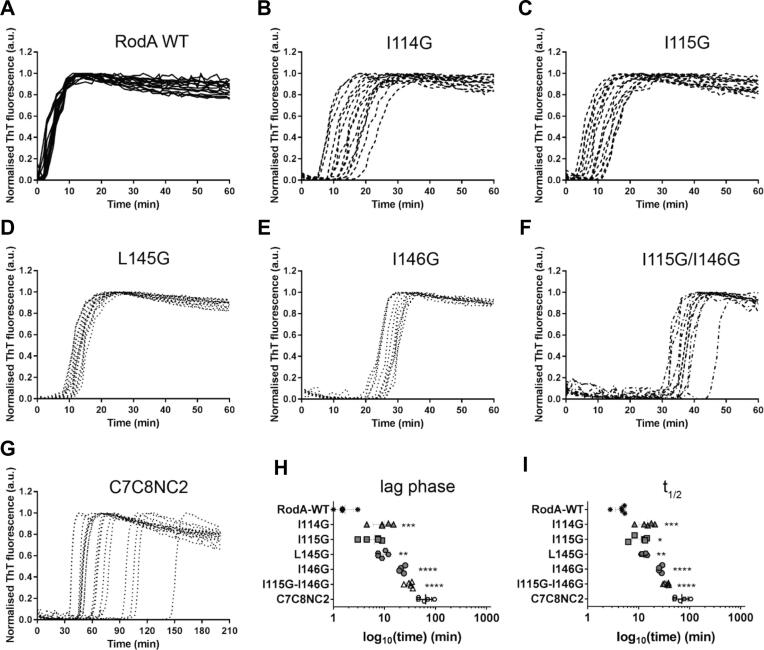
The effect of mutation(s) on the kinetics of RodA rodlet self-assembly assessed by Thioflavin T fluorescence at 50 °C. Amyloid assembly profiles for (A) RodA WT; single point mutants (B) RodA I114G, (C) RodA I115G, (D) RodA L145G and (E) RodA I146G; (F) double point mutant RodA I115G/I146G and (G) C7C8NC2 chimera. (H) Lag time and (I) time taken to reach half maximum ThT fluorescence. (See the material and methods section for calculation of lag time and time to reach half maximum).

Analysis of the RodA sequence by the consensus method Amylpred2 (Tsolis et al., 2013) indicated that stretches of residues (112–117 and 143–147) within the C4-C5 and C7-C8 inter-cysteine regions are predicted as amyloidogenic and could potentially participate in the cross β-sheets at the core of amyloid fibres. To investigate the role of these sequences in the context of the intact protein and interface-driven self-assembly, a number of mutations were introduced into rRodA. Individual or multiple glycine and serine substitutions of isoleucine and leucine residues were used to reduce side chain hydrophobicity and size and to introduce flexibility or polarity at sites within the protein structure. The kinetics of self-assembly of rRodA from the monomeric form into the amyloid rodlet form were monitored by changes in fluorescence of the amyloid binding dye Thioflavin T (ThT) during *in vitro* self-assembly assays (Fig. 2).

Only mutations to the rRodA sequence in the C4-C5 loop or in the C7-C8 loop resulted in a delay in self-assembly of rRodA mutants, relative to wild-type (WT) rRodA. Single-residue mutations introduced a significant lag phase of 10–20 min into the self-assembly profile, whereas no lag phase was detected for WT rRodA under these conditions (Fig. 2). The effect of double mutations in the C4–C5 and C7–C8 loops was additive since a rRodA carrying the double slowing mutations I115G/I146G displayed a longer lag phase than rRodA I115G and rRodA I146G (Fig. 2). Mutations introduced in other regions of rRodA outside of the C4–C5 and C7–C8 loops did not have an effect on RodA self-assembly (data not shown). Strikingly, a chimeric protein rRodA C7C8NC2, which has the central sequence of the rRodA C7–C8 loop replaced with the C7–C8 loop from the non-amyloidogenic class II hydrophobin NC2, was able to assemble into ThT-positive structures (Fig. 2). A similar chimera in which the NC2 region was inserted into the hydrophobin EAS did not produce ThT-positive rodlets. These data show that in contrast to the hydrophobin EAS where only the C7–C8 loop is required for rodlet formation (Macindoe et al., 2012), RodA rodlet assembly involves the C4–C5 and the C7–C8 loops.

**Fig. 3:**
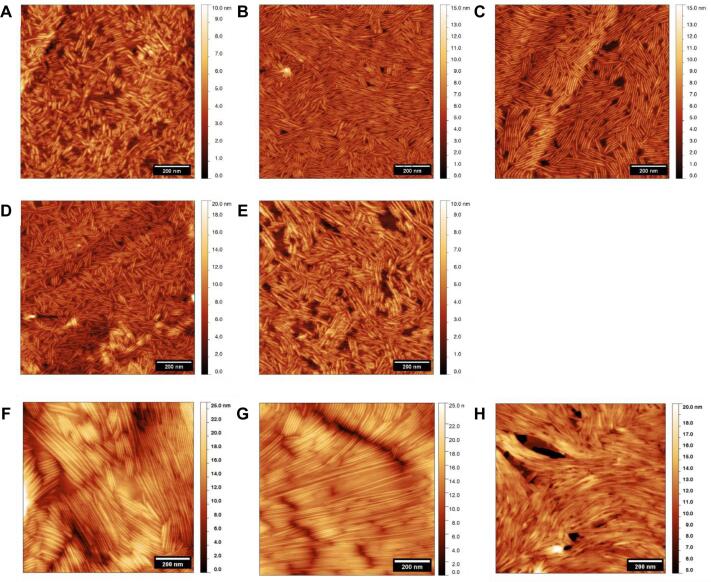
Assembly of RodA WT and mutant proteins on highly oriented pyrolytic graphite. Atomic force micrographs showing the surface morphology of rodlets formed by (A) RodA WT, (B) RodA I114G, (C) RodA I115G, (D) RodA L145G and (E) RodA I146G after incubation at room temperature on graphite surfaces. Surface layers containing rodlets formed by (F) RodA WT, (G) double mutant RodA I115G/I146G and (H) C7C8NC2 chimera after incubation at 50 °C for 2 h. (See the Material and Methods section for the AFM experimental conditions).

As revealed by atomic force microscopy (AFM) images in Fig. 3A, WT rRodA rodlets have comparable dimensions to those reported for other class I hydrophobins: width of 10.1 ± 1.8 nm and height of 2.0 ± 0.5 nm (Table S2). Introduction of the single mutations between cysteines 4 and 5 (Fig. 3 B,C) and cysteines seven and eight (Fig. 3 D,E) that slowed assembly kinetics did not grossly alter the morphology of the final assembled rodlet structure on a hydrophobic graphite surface (HOPG), as probed by AFM. In contrast, the rRodA protein carrying the double mutation and the rRodA C7C8NC2 chimera did not spontaneously form rodlets when dried onto HOPG at room temperature. However, when rRodA I115G/I146G protein was incubated at 50 °C for 2 h on the graphite surface, it formed a film composed of very long rodlets, as did WT rRodA assembled at this temperature (Fig. 3F, G). The rRodA C7C8NC2 chimera also formed rodlets only when prepared at 50 °C. However, the surface details and lateral packing of the rodlets formed by the chimera were less distinct than observed with WT rRodA rodlets (Fig. 3H).

**Fig. 4:**
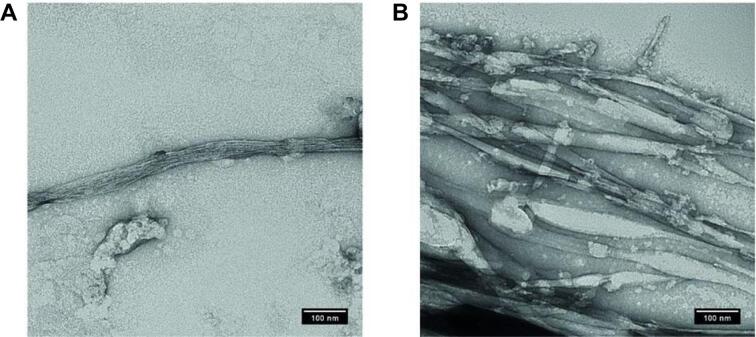
Transmission electron micrographs of fibrillar material formed by (A) PIIGIPIQDL, a peptide derived from the C4-C5 region of RodA and (B) SLIGL, a peptide with predicted amyloidogenic sequence from the C7-C8 region.

The results obtained with the different point mutated rRodA proteins suggest that while a single amyloidogenic region can support the incorporation of monomers into a fibrillar structure albeit with slower kinetics, the absence of a second interaction within the rodlets causes a morphological disturbance, with consequences for the organization and lateral interactions between rodlets within the film. The self-assembling nature of the sequences between the 4-5th and 7-8th cysteine residues was confirmed by analysis of peptides containing these sequences. Transmission electron microscopy showed that both the peptide PIIGIPIQDL (Fig. 4A) that originates from the C4-C5 region and SLIGL (Fig. 4B) found in the C7-C8 region can form fibrillar structures. In PIIGIPIQDL, fibrillar aggregation occurs at a concentration of 200 µg/ml, with many smaller fibrils bundling together to form a single fibril. The SLIGL peptide formed thicker single fibrils with a crystalline nature at 200 µg/ml.

### 2.3 Assembly of the rodlet structure on the conidium

**Fig. 5:**
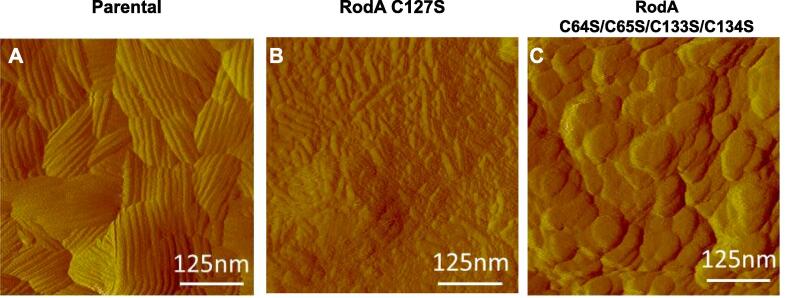
Cysteine mutations impair the presence of rodlets at the conidial surface. AFM deflection images showing the structure of conidial surface imaged in liquid conditions for: (A) the parental, (B) one cysteine (C127S) and (C) four cysteine (C64S/C65S/C133S/C134S) mutants.

**Fig. 6:**
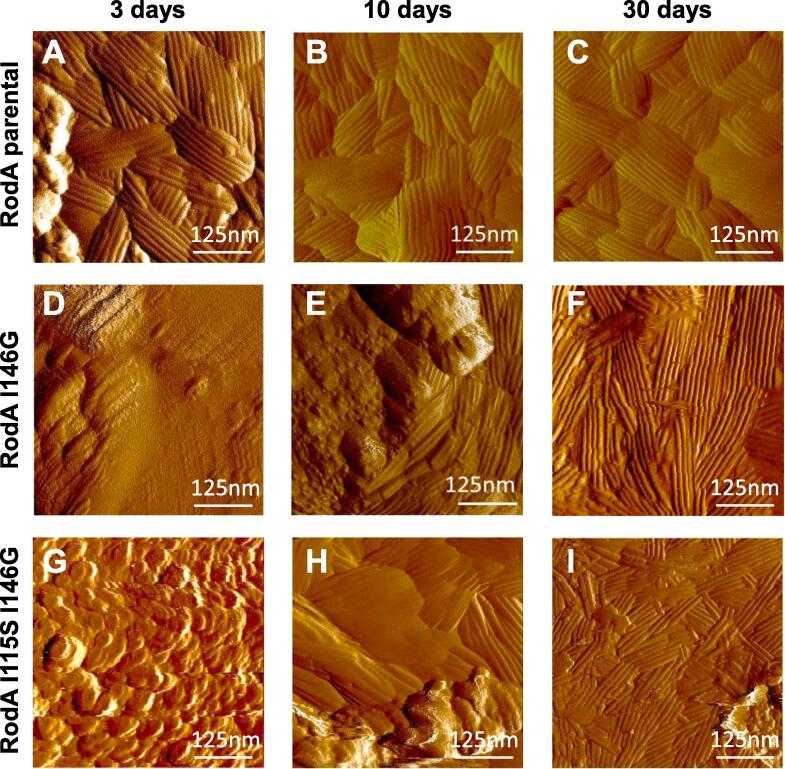
Delay in rodlets appearance on the surface of conidia imaged in liquid conditions. AFM deflection images of the rodlets layer for (A-C) the parental, (D-F) I146G and (G-I) I115S/I146G mutants after 3 (A, D, G), 10 (B, E, H) and 30 (C, F, I) days of growth.

**Fig. 7:**
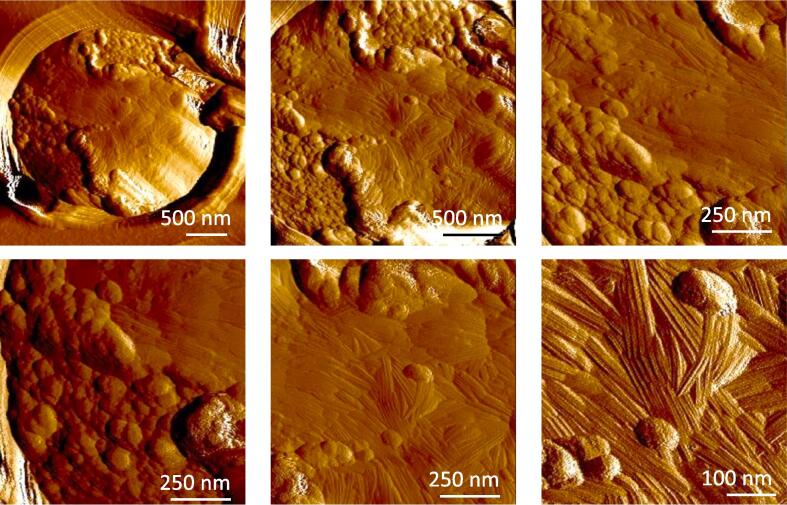
The surface of the RodA I115S/I146G double mutant is only partially covered by rodlets. AFM deflections images (at different magnification) representative of the conidium surface of the RodA I115S/I146G double mutant after 30 days of culture with different magnifications.

In order to determine the effect of RodA mutations on conidial rodlet assembly, different mutations were also introduced into the *RODA* gene (Figs. S4 and S5). The effect of these mutations on the rodlet structure on the conidial surface was investigated with AFM (Fig. 5, Fig. 6, Fig. 7). Incubation of parental strain conidia with hydrofluoric acid (HF) or formic acid (FA) to isolate native RodA always results in the isolation of two protein bands (Aimanianda et al., 2009). MS, N-terminal sequencing and NMR data led to the identification of both molecular species. The higher Mr band corresponds to the full-length protein without the signal peptide with an N-terminal pyroglutamic acid (PCA) arising from a spontaneous deamidation of the N-terminal Gln residue at position 21 (PCA21-L159). The lower Mr species corresponds to the F41-L159 peptide, missing 40 amino acids from the N-terminus. The mutant with a RodA protein corresponding to the band with lower Mr seen in conidial extracts did not show any alteration in rodlet formation on the surface of the conidium (Fig. S4). This result indicates that the N-terminal region, which is unstructured in the monomer, does not play a role in the assembly of the rodlet layer.

The role of cysteine bridges in rodlet formation has been characterized earlier in *S. commune* and *M. grisea* (de Vocht et al., 2000, Kershaw et al., 2005). Herein, we evaluated the necessity for intact disulfide bridges within RodA for correct rodlet assembly in *A. fumigatus* by the construction and characterization of mutants with the *RODA* gene mutated in one cysteine (C127S) or in four of them (C64S/C65S/C133S/C134S) to disrupt one or the four RodA disulfide bridges. None of these mutants produced rodlets on the conidial surface as shown by AFM (Fig. 5). Moreover, negative Western blots of the formic acid extracts of these cysteine mutants with an anti-RodA antibody demonstrated that RodA was not secreted to the conidial surface (Fig. S6).

*A. fumigatus* mutants harboring the same RodA point mutations as the ones undertaken with the recombinant protein (I114G, I115S, L145S, I146G and I115S/I146G) were generated. Like in the parental strain, RodA could be detected on SDS-PAGE as doublets after HF or FA extraction from all these RodA loop region mutants (Fig. S7). The surface organization of mutants resulting from the point mutations I114G, I115S, L145S, I146G and I115S/I146G was investigated by AFM. The hydrophobicity of the rodlet structure of the RodA I114G, RodA I115S and RodA L145S mutants was not affected, as seen by the adhesion force histograms obtained using chemical force microscopy with hydrophobic AFM tips (Dague et al., 2008b, Dague et al., 2007), data not shown]. In agreement with the ThT data obtained with mutated rRodA in *in vitro* assays (Fig. 2), the single-point mutation I146G and the double mutation I115S/I146G, which both led to a longer lag phase, affected the assembly of the rodlet layer on the surface of the conidium (Fig. 6). The appearance of the rodlets on the surface of the conidium was dependent on the conidial age. At 3-days of growth, conidia of these two mutants did not show any rodlets on their surface unlike parental conidia; however, the number of rodlets increased with the age of the conidia. The maximal rodlet formation was seen after 30-days of culture without further evolution of the rodlet morphology afterwards. Interestingly, two modifications of the rodlet structure were seen on the mutant conidia: (1) the length of the rodlets significantly increased from 147 nm observed for the parental to 270 and 234 nm for the I146G and I115S/I146G mutants, respectively (Fig. S8); (2) while parental rodlets coated the entire conidium, the coverage of the conidium surface by the mutated rodlets was incomplete, with a coverage ranging from 20 to 50% of the conidial surface for the I115S/I146G mutant (Fig. 7).

In addition, the surfaces of the conidia of the I146G and I115S/I146G mutants were covered at least partially by an amorphous material. MS analysis of the extract obtained after incubation of the conidia in formic acid for 2 h indicated that over 1200 proteins were quantified in the I115S/I146G mutant conidia and this number greatly exceed the number of proteins detected with its parental strain, demonstrating the presence of a much higher concentration of proteins in the mutant cell wall (Data are available via ProteomeXchange with the identifier PXD008503). In spite of differences seen with AFM, a comparable number of proteins were extracted from 10-days or 30-day old I115S/I146G mutant conidia (1399 and 1278 respectively). The identification of the mutated and wild type RodA protein in the extracts from the mutant and parental strains validated the MS experiment. Analysis of proteins with a signal peptide, which are specifically targeted to be secreted to the cell wall, showed that 56 and 68 proteins were present in the cell wall of the 10 and 30-days old conidia, respectively (Table 1). Among the 31 proteins extracted from both 10- and 30-days mutant conidia, some of them such as disulfide isomerases, carboxypeptidases or phiA (Melin et al., 2003, Monod et al., 2002) could be specifically involved in the maturation of RodA. Others such as glucanases, chitinases, transglycosidases or GPI proteins are known to be involved in cell wall modifications (Latgé et al., 2017). Among these proteins, several (Sun1, Crf1, MP1, ChiB1, Aspf4, DPPV) are also known as antigens/allergens in agreement with their cell wall surface localization (Beauvais et al., 1997, Gastebois et al., 2013, Latgé, 1999). The coating of the outer conidial layer by an amorphous material was confirmed by the positive labelling of the conidia of these two mutants with the wheat germ agglutinin and concanavalin A lectins, in contrast with the parental strain (Fig. S9). The hydrophilicity of the I115S/I146G double mutant conidia seen by AFM was due to an incomplete coverage of the conidial surface by the rodlets and to the presence of amorphous (glyco)proteins on the surface of the mutant cell wall. Accordingly, the I115S/I146G mutant conidial colonies appeared black and hydrophilic like the Δ*rodA* mutant, as did (but to a lesser extent) the conidia from the single I146G mutant (Fig. S10). Complementation of the mutated *RODA* gene with the wild type gene led to the production of normal hydrophobic conidia displaying the characteristic green color of the parental strain instead of the black color of the mutant conidia (Fig. S10).

**Table 1:** Signal-peptide-containing proteins extracted with 2 h incubation in formic acid. Only proteins that are specific of the double mutant conidia RodA I115S/I146G (and absent from the Ku80 parental strain) are listed. **(A)** 30 days old conidia and **(B)** 10 days old conidia. The most abundant proteins present in both 30 and 10 days old conidia are listed in **(C)**. Proteins are ranked in decreasing order of abundancy based on iBAQ (intensity Based Absolute Quantification) values. The mean iBAQ is calculated from the three biological replicates. Over 1000 proteins were identified and quantified in the formic acid extracts from each strain and were deposited in the PRIDE data base PXD008503 (http://proteomecentral.proteomexchange.org/cgi/GetDataset).

A	Signal-peptide-containing proteins extracted with 2h in formic acid from 30d old conidia, present in I115S/I146G and absent in parental strain									
Gene ID	BLAST Af293	UniProt	Description	Present in I115S/I146G, absent in parental, 10d	Score of SignalP	Unique sequence coverage (%)	Protein length	MW (kDa)	Mean iBAQ I115S/I146G 30d	Mean Unique Peptides I115S/I146G 30d
AFUB_086950	AFUA_7G00370	B0YBK6	Uncharacterized protein	Yes	0.78	29.30	198	21.384	17579000	4.0
AFUB_097339	AFUA_6G00180	B0YDZ8	Putative uncharacterized protein	No	0.74	38.50	109	12.053	17425867	2.7
AFUB_096860	AFUA_6G00610	B0YDU9	Putative uncharacterized protein	No	0.85	21.40	98	10.853	8641800	1.0
AFUB_060890	AFUA_5G13180	B0Y1U6	Agmatinase, putative	Yes	0.68	28.40	391	42.179	7932333	6.0
AFUB_059890	AFUA_5G12260	B0Y1D4	Disulfide isomerase (TigA), putative	Yes	0.87	54.60	368	40.253	5965500	14.3
AFUB_064740	AFUA_4G07650	B0Y5V8	Peptidyl-prolyl cis-trans isomerase	No	0.92	45.00	209	22.961	4038867	6.0
AFUB_072730	AFUA_6G06800	B0Y766	Probable carboxypeptidase	Yes	0.58	25.90	440	46.377	3466967	5.7
AFUB_083440	AFUA_8G04120	B0YA52	Carboxypeptidase	No	0.56	28.90	551	61.278	2966367	8.7
AFUB_059210	AFUA_5G11640	B0Y110	Secretory pathway protein Ssp120, putative	Yes	0.64	36.00	203	23.277	2893100	3.7
AFUB_070080	AFUA_4G13190	B0Y4S2	Endosomal cargo receptor (Erv25), putative	Yes	0.93	33.80	266	30.039	2381567	4.3
AFUB_091030	AFUA_7G05450	B0YCQ5	SUN domain protein (Uth1), putative	Yes	0.50	23.20	414	43.504	2110200	5.3
AFUB_064480	AFUA_4G07390	B0Y5T1	Endosomal cargo receptor (Erp5), putative	No	0.90	20.80	231	25.863	1582933	3.0
AFUB_066060	AFUA_4G08960	B0Y688	GPI anchored cell wall protein, putative	No	0.51	11.90	386	39.611	1522767	2.3
AFUB_076030	AFUA_6G09980	B0Y842	Patched sphingolipid transporter (Ncr1), putative	Yes	0.66	4.60	1273	140.400	1482511	2.3
AFUB_038670	AFUA_3G10490	B0XXV2	DNA damage response protein (Dap1), putative	Yes	0.69	37.40	155	17.221	1454907	3.0
AFUB_015530	AFUA_1G16190	B0XNL0	Extracellular cell wall glucanase Crf1/allergen Asp F9	Yes	0.77	22.50	395	40.269	1212400	4.0
AFUB_031800	AFUA_2G16120	B0XUX8	Translocon-associated protein, alpha subunit, putative	Yes	0.84	42.10	261	28.158	1169660	2.3
AFUB_010690	AFUA_1G11260	B0XQP5	Uncharacterized protein	No	0.91	56.70	97	10.186	1167077	1.0
AFUB_002360	AFUA_1G01980	B0XRG2	IgE binding protein, putative	Yes	0.51	12.30	179	18.514	1161590	1.7
AFUB_047560	AFUA_3G00840	B0XX33	FAD-dependent oxygenase, putative	No	0.59	17.60	507	55.026	921023	5.7
AFUB_045140	AFUA_3G03080	B0Y002	Endo-1,3(4)-beta-glucanase, putative	No	0.90	21.40	285	31.087	869887	3.0
AFUB_043810	AFUA_3G04160	B0XZB5	Ser/Thr protein phosphatase family	Yes	0.51	14.40	626	71.262	860230	4.3
AFUB_010920	AFUA_1G11490	B0XQR8	Endopolyphosphatase	Yes	0.78	3.40	668	76.206	846930	2.7
AFUB_020900	AFUA_2G03830	B0XUQ5	Allergen Asp F4	Yes	0.50	25.20	322	34.093	784443	2.7
AFUB_090580	AFUA_7G05015	B0YCL1	Glyoxylase family protein, putative	Yes	0.56	14.70	225	25.273	746237	2.7
AFUB_061840	AFUA_5G14150	B0Y2H6	Lipase, putative	No	0.63	4.00	470	51.907	731363	1.0
AFUB_083670	AFUA_8G03905	B0YA75	Uncharacterized protein	Yes	0.57	9.60	415	45.097	689597	1.7
AFUB_019400	AFUA_2G02310	B0XTX2	Cortical patch protein SUR7, putative	Yes	0.82	6.70	239	26.609	670723	1.0
AFUB_051770	AFUA_5G03260	B0Y2S3	Endosomal cargo receptor (Erp3), putative	No	0.81	31.50	241	27.159	641977	2.7
AFUB_009000	AFUA_1G09550	B0XQ77	Dynein light chain (Tctex1), putative	Yes	0.53	21.70	143	15.325	639187	2.0
AFUB_010900	AFUA_1G11470	B0XQR6	Endosomal cargo receptor (P24), putative	Yes	0.78	27.10	218	25.056	590885	2.0
AFUB_069760	AFUA_4G12850	B0Y4P0	Calnexin	No	0.54	25.00	563	61.853	576790	4.7
AFUB_097340	AFUA_6G00160	B0YDZ9	Ser/Thr protein phosphatase family protein	Yes	0.73	23.90	666	75.547	565267	4.7
AFUB_038220	AFUA_3G10910	B0XXQ4	Glutaminase, putative	Yes	0.65	17.90	834	92.757	548343	6.0
AFUB_080680	AFUA_8G07120	B0Y9D8	Beta-1,6-glucanase, putative	No	0.55	23.60	488	51.430	533200	2.7
AFUB_024700	AFUA_2G08790	B0XRS8	Putative metallocarboxypeptidase ecm14	Yes	0.95	6.70	586	66.068	528503	2.3
AFUB_034540	AFUA_3G14680	B0XZV8	Lysophospholipase 3	No	0.81	14.00	630	67.416	525853	4.3
AFUB_028320	AFUA_2G12680	B0XSZ4	Uncharacterized protein	No	0.84	14.10	185	19.585	504547	2.0
AFUB_017890	AFUA_2G00810	B0XT37	Purine nucleoside permease, putative	No	0.83	9.90	403	43.329	465497	1.7
AFUB_015760	AFUA_1G16420	B0XNV1	GPI anchored protein, putative	No	0.58	3.10	523	58.528	379147	1.0
AFUB_092390	AFUA_7G06810	B0YBD2	L-amino acid oxidase LaoA	No	0.65	10.20	697	78.612	352340	3.0
AFUB_056510	AFUA_5G08970	B0Y449	Oligosaccharyl transferase subunit (beta), putative	Yes	0.94	16.90	462	51.642	331680	2.3
AFUB_061470	AFUA_5G13730	B0Y269	NlpC/P60-like cell-wall peptidase, putative	Yes	0.81	12.30	359	38.763	323073	2.3
AFUB_100370	AFUA_4G02780	B0YEU1	Toxin biosynthesis peroxidase, putative	No	0.83	11.10	324	36.980	311520	0.7
AFUB_014800	AFUA_1G15250	B0XN71	Autophagy protein Atg27, putative	Yes	0.76	8.40	333	37.377	307030	1.0
AFUB_101570	AFUA_4G01070	B0YF50	Acid phosphatase, putative	No	0.74	11.90	293	30.459	304120	1.7
AFUB_021510	AFUA_2G04480	B0XV35	Xylosidase/arabinosidase, putative	No	0.81	19.10	371	40.803	289893	2.0
AFUB_030040	AFUA_2G14420	B0XU21	Cutinase	No	0.66	7.90	366	35.677	269740	0.7
lap1	lap1	B0YED6	Leucine aminopeptidase 1	No	0.84	13.90	388	43.127	267110	2.0
AFUB_085200	AFUA_8G01410	B0YAM7	Class V chitinase ChiB1	Yes	0.76	9.00	433	47.622	245500	0.7
AFUB_027870	AFUA_2G12180	B0XSP1	Lectin family integral membrane protein, putative	No	0.72	16.20	327	36.197	244833	2.0
AFUB_052800	AFUA_5G04280	B0Y335	Uncharacterized protein	Yes	0.83	16.70	245	26.115	229443	2.3
AFUB_034660	AFUA_3G14570	B0XZX0	Histidine acid phosphatase, putative	No	0.74	7.10	462	51.295	213797	2.0
btgE	btgE	B0Y9Q9	Probable beta-glucosidase btgE	No	0.49	5.80	565	58.147	200803	1.3
AFUB_052440	AFUA_5G03940	B0Y2Z9	Mutanase	No	0.89	15.00	446	49.981	190812	1.3
AFUB_045510	0	B0Y037	Uncharacterized protein	No	0.68	23.60	229	23.817	186013	1.0
AFUB_089550	AFUA_7G04020	B0YCB0	Lipase, putative	No	0.64	5.00	463	49.258	182750	0.7
AFUB_003170	AFUA_1G02780	B0XML5	L-asparaginase	No	0.79	4.70	379	39.784	181210	1.0
AFUB_065820	AFUA_4G08720	B0Y665	Lysophospholipase 1	No	0.78	4.60	633	68.143	179210	2.0
AFUB_032470	AFUA_2G16800	B0XVA9	Lectin family integral membrane protein, putative	Yes	0.69	17.40	328	36.070	167933	2.0
AFUB_036000	AFUA_3G13200	B0XWI1	1,3-beta-glucanosyltransferase	No	0.72	4.10	460	49.933	135095	1.0
AFUB_039870	AFUA_3G09250	B0XY72	Cell wall glucanase, putative	Yes	0.93	6.30	363	40.590	107533	1.0
AFUB_100920	AFUA_4G00390	B0YEY4	Glycosyl hydrolase, putative	No	0.66	7.80	883	98.929	93101	3.3
AFUB_020340	AFUA_2G03270	B0XUD4	Glycosyl hydrolase, putative	No	0.80	2.10	620	69.305	84869	1.0
AFUB_052020	AFUA_5G03500	B0Y2U6	Alpha glucosidase II, alpha subunit, putative	No	0.77	10.50	967	109.650	80189	2.0
AFUB_030100	AFUA_2G14480	B0XU27	Oxidoreductase, FAD-binding, putative	No	0.89	4.20	473	50.494	68197	0.7
AFUB_067180	AFUA_4G10070	B0Y6J5	Alpha-1,2-Mannosidase	Yes	0.64	1.60	641	73.606	39384	1.0
AFUB_046050	AFUA_3G02280	B0Y0F9	Alpha,alpha-trehalose glucohydrolase TreA/Ath1	No	0.81	1.20	1072	117.000	29926	0.7

B	Signal-peptide-containing proteins extracted with 2h in formic acid from 10d old conidia, present in I115S/I146G and absent in parental strain									
Gene ID	BLAST Af293	UniProt	Description	Present in I115S/I146G, absent in parental 30d	Score of SignalP	Unique sequence coverage (%)	Protein length	MW (kDa)	Mean iBAQ I115S/I146G 10d	Mean Unique Peptides I115S/I146G 10d
AFUB_046180	AFUA_3G02190	B0Y0H2	Uncharacterized protein	No	0.87	30.30	297	33.610	7462267	8.7
AFUB_059890	AFUA_5G12260	B0Y1D4	Disulfide isomerase (TigA), putative	Yes	0.87	54.60	368	40.253	4837900	14.0
AFUB_031800	AFUA_2G16120	B0XUX8	Translocon-associated protein, alpha subunit, putative	Yes	0.84	42.10	261	28.158	4398400	4.0
AFUB_070080	AFUA_4G13190	B0Y4S2	Endosomal cargo receptor (Erv25), putative	Yes	0.93	33.80	266	30.039	3296267	5.3
AFUB_038670	AFUA_3G10490	B0XXV2	DNA damage response protein (Dap1), putative	Yes	0.69	37.40	155	17.221	3025733	4.7
AFUB_059210	AFUA_5G11640	B0Y110	Secretory pathway protein Ssp120, putative	Yes	0.64	36.00	203	23.277	2806833	4.7
AFUB_045170	AFUA_3G03060	B0Y004	Cell wall protein phiA	No	0.58	37.80	185	19.380	2437133	3.0
AFUB_038220	AFUA_3G10910	B0XXQ4	Glutaminase, putative	Yes	0.65	17.90	834	92.757	2213600	12.3
AFUB_087640	AFUA_7G01060	B0YBS2	Cysteine-rich secreted protein	No	0.81	22.40	343	36.860	2169400	3.7
AFUB_072730	AFUA_6G06800	B0Y766	Probable carboxypeptidase	Yes	0.58	25.90	440	46.377	1894433	3.7
AFUB_071360	AFUA_4G14205	B0Y546	Uncharacterized protein	No	0.88	8.00	138	14.405	1737567	1.0
AFUB_046170	AFUA_3G02200	B0Y0H1	Uncharacterized protein	No	0.89	17.20	192	20.681	1636300	2.7
AFUB_060890	AFUA_5G13180	B0Y1U6	Agmatinase, putative	Yes	0.68	28.40	391	42.179	1631667	3.3
AFUB_010900	AFUA_1G11470	B0XQR6	Endosomal cargo receptor (P24), putative	Yes	0.78	27.10	218	25.056	1595133	4.7
AFUB_097340	AFUA_6G00160	B0YDZ9	Ser/Thr protein phosphatase family protein	Yes	0.73	23.90	666	75.547	1560833	10.0
AFUB_091030	AFUA_7G05450	B0YCQ5	SUN domain protein (Uth1), putative	Yes	0.50	23.20	414	43.504	1474467	5.7
AFUB_099880	AFUA_4G03240	B0YEP2	Cell wall serine-threonine-rich galactomannoprotein Mp1	No	0.83	33.50	284	27.361	1119097	3.7
AFUB_056510	AFUA_5G08970	B0Y449	Oligosaccharyl transferase subunit (beta), putative	Yes	0.94	16.90	462	51.642	994130	4.3
AFUB_009000	AFUA_1G09550	B0XQ77	Dynein light chain (Tctex1), putative	Yes	0.53	21.70	143	15.325	865887	2.0
AFUB_086950	AFUA_7G00370	B0YBK6	Uncharacterized protein	Yes	0.78	29.30	198	21.384	790777	1.0
AFUB_020580	AFUA_2G03510	B0XUM7	Carboxypeptidase	No	0.84	23.00	534	59.725	774907	2.7
bglF	bglF	B0Y7Q8	Probable beta-glucosidase F	No	0.79	42.70	869	93.059	764553	7.3
AFUB_083670	AFUA_8G03905	B0YA75	Uncharacterized protein	Yes	0.57	9.60	415	45.097	761603	3.0
AFUB_022820	AFUA_2G05790	B0XVU7	Oligosaccharyl transferase subunit (alpha), putative	No	0.78	20.80	504	56.149	717993	4.3
AFUB_090580	AFUA_7G05015	B0YCL1	Glyoxylase family protein, putative	Yes	0.56	14.70	225	25.273	676803	1.7
AFUB_024920	AFUA_2G09030	B0XRV0	Dipeptidyl-peptidase 5	No	0.74	16.00	721	79.742	589393	7.3
AFUB_032470	AFUA_2G16800	B0XVA9	Lectin family integral membrane protein, putative	Yes	0.69	17.40	328	36.070	540273	3.7
AFUB_002360	AFUA_1G01980	B0XRG2	IgE binding protein, putative	Yes	0.51	12.30	179	18.514	538723	1.3
AFUB_019400	AFUA_2G02310	B0XTX2	Cortical patch protein SUR7, putative	Yes	0.82	6.70	239	26.609	516630	1.0
AFUB_039870	AFUA_3G09250	B0XY72	Cell wall glucanase, putative	Yes	0.93	6.30	363	40.590	514650	2.0
AFUB_063770	AFUA_4G06700	B0Y5L1	GPI anchored cell wall protein, putative	No	0.81	5.90	221	22.620	485620	1.7
AFUB_043810	AFUA_3G04160	B0XZB5	Ser/Thr protein phosphatase family	Yes	0.51	14.40	626	71.262	377700	1.3
AFUB_029830	AFUA_2G14210	B0XTT1	Mitochondrial dihydroxy acid dehydratase, putative	No	0.51	12.60	642	68.256	371027	5.0
AFUB_098310	AFUA_4G04690	B0YE87	Uncharacterized protein	No	0.73	18.70	209	22.594	333057	1.0
AFUB_025060	AFUA_2G09180	B0XRW4	Coatomer subunit zeta, putativ	No	0.48	13.90	201	21.929	332405	0.7
AFUB_024700	AFUA_2G08790	B0XRS8	Putative metallocarboxypeptidase ecm14	Yes	0.95	6.70	586	66.068	308810	0.7
AFUB_014610	AFUA_1G15050	B0XN52	Hsp70 family chaperone Lhs1/Orp150, putative	No	0.58	10.00	997	108.750	300040	5.3
AFUB_010920	AFUA_1G11490	B0XQR8	Endopolyphosphatase	Yes	0.78	3.40	668	76.206	295473	2.0
AFUB_052800	AFUA_5G04280	B0Y335	Uncharacterized protein	Yes	0.83	16.70	245	26.115	293027	2.3
AFUB_023360	AFUA_2G06280	B0XW28	Oligosaccharyl transferase subunit (Gamma), putative	No	0.69	7.10	325	35.939	260640	0.7
AFUB_056810	AFUA_5G09270	B0Y479	Uncharacterized protein	No	0.87	6.20	952	103.550	223923	4.0
AFUB_070260	AFUA_4G13340	B0Y4T7	DUF907 domain protein	No	0.93	3.90	721	78.538	178277	2.0
AFUB_061470	AFUA_5G13730	B0Y269	NlpC/P60-like cell-wall peptidase, putative	Yes	0.81	12.30	359	38.763	169897	1.3
AFUB_085200	AFUA_8G01410	B0YAM7	Class V chitinase ChiB1	Yes	0.76	9.00	433	47.622	159987	1.0
AFUB_074470	AFUA_6G08510	B0Y7N9	Cell wall glucanase, putative	No	0.79	10.30	436	45.674	152803	1.0
AFUB_005670	AFUA_1G05320	B0XP24	Disulfide isomerase, putative	No	0.87	7.50	478	51.299	146322	2.3
AFUB_020900	AFUA_2G03830	B0XUQ5	Allergen Asp F4	Yes	0.50	25.20	322	34.093	138837	1.3
AFUB_061010	AFUA_5G13300	B0Y1V8	Aspartic protease pep1	No	0.69	33.20	395	41.613	133245	0.7
AFUB_014800	AFUA_1G15250	B0XN71	Autophagy protein Atg27, putative	Yes	0.76	8.40	333	37.377	121900	1.3
AFUB_079260	AFUA_1G01540	B0Y912	Endonuclease/exonuclease/phosphatase family protein	No	0.59	9.30	439	47.788	100628	1.0
AFUB_015530	AFUA_1G16190	B0XNL0	Extracellular cell wall glucanase Crf1/allergen Asp F9	Yes	0.77	22.50	395	40.269	84105	1.0
AFUB_076030	AFUA_6G09980	B0Y842	Patched sphingolipid transporter (Ncr1), putative	Yes	0.66	4.60	1273	140.400	81393	2.0
AFUB_035150	AFUA_3G14060	B0Y088	Palmitoyl-protein thioesterase	No	0.59	5.40	333	37.495	80447	1.0
AFUB_024270	AFUA_2G08300	B0XWB9	DnaJ domain protein, putative	No	0.94	4.20	427	48.528	75414	0.7
AFUB_026370	AFUA_2G10590	B0XS92	Disulfide isomerase, putative	No	0.88	4.70	737	82.846	58610	0.7
AFUB_067180	AFUA_4G10070	B0Y6J5	Alpha-1,2-Mannosidase	Yes	0.64	1.60	641	73.606	57328	1.0

C	Signal-peptide-containing proteins extracted with 2h in formic acid from 10d and 30d old conidia, present in I115S/I146G and absent in parental strain									
Gene ID	Blast Af293	UniProt	Description	Present in 115146, absent in parental of 30d	Score of SignalP	Unique sequene coverage (%)	Protein length	MW (kDa)	Mean iBAQ I115S/I146G 10d	Mean Unique Peptides I115S/I146G 10d
AFUB_059890	AFUA_5G12260	B0Y1D4	Disulfide isomerase (TigA), putative	Yes	0.87	54.60	368	40.253	4837900	14.0
AFUB_031800	AFUA_2G16120	B0XUX8	Translocon-associated protein, alpha subunit, putative	Yes	0.84	42.10	261	28.158	4398400	4.0
AFUB_070080	AFUA_4G13190	B0Y4S2	Endosomal cargo receptor (Erv25), putative	Yes	0.93	33.80	266	30.039	3296267	5.3
AFUB_038670	AFUA_3G10490	B0XXV2	DNA damage response protein (Dap1), putative	Yes	0.69	37.40	155	17.221	3025733	4.7
AFUB_059210	AFUA_5G11640	B0Y110	Secretory pathway protein Ssp120, putative	Yes	0.64	36.00	203	23.277	2806833	4.7
AFUB_038220	AFUA_3G10910	B0XXQ4	Glutaminase, putative	Yes	0.65	17.90	834	92.757	2213600	12.3
AFUB_072730	AFUA_6G06800	B0Y766	Probable carboxypeptidase	Yes	0.58	25.90	440	46.377	1894433	3.7
AFUB_060890	AFUA_5G13180	B0Y1U6	Agmatinase, putative	Yes	0.68	28.40	391	42.179	1631667	3.3
AFUB_010900	AFUA_1G11470	B0XQR6	Endosomal cargo receptor (P24), putative	Yes	0.78	27.10	218	25.056	1595133	4.7
AFUB_097340	AFUA_6G00160	B0YDZ9	Ser/Thr protein phosphatase family protein	Yes	0.73	23.90	666	75.547	1560833	10.0
AFUB_091030	AFUA_7G05450	B0YCQ5	SUN domain protein (Uth1), putative	Yes	0.50	23.20	414	43.504	1474467	5.7
AFUB_056510	AFUA_5G08970	B0Y449	Oligosaccharyl transferase subunit (beta), putative	Yes	0.94	16.90	462	51.642	994130	4.3
AFUB_009000	AFUA_1G09550	B0XQ77	Dynein light chain (Tctex1), putative	Yes	0.53	21.70	143	15.325	865887	2.0
AFUB_086950	AFUA_7G00370	B0YBK6	Uncharacterized protein	Yes	0.78	29.30	198	21.384	790777	1.0
AFUB_083670	AFUA_8G03905	B0YA75	Uncharacterized protein	Yes	0.57	9.60	415	45.097	761603	3.0
AFUB_090580	AFUA_7G05015	B0YCL1	Glyoxylase family protein, putative	Yes	0.56	14.70	225	25.273	676803	1.7
AFUB_032470	AFUA_2G16800	B0XVA9	Lectin family integral membrane protein, putative	Yes	0.69	17.40	328	36.070	540273	3.7
AFUB_002360	AFUA_1G01980	B0XRG2	IgE binding protein, putative	Yes	0.51	12.30	179	18.514	538723	1.3
AFUB_019400	AFUA_2G02310	B0XTX2	Cortical patch protein SUR7, putative	Yes	0.82	6.70	239	26.609	516630	1.0
AFUB_039870	AFUA_3G09250	B0XY72	Cell wall glucanase, putative	Yes	0.93	6.30	363	40.590	514650	2.0
AFUB_043810	AFUA_3G04160	B0XZB5	Ser/Thr protein phosphatase family	Yes	0.51	14.40	626	71.262	377700	1.3
AFUB_024700	AFUA_2G08790	B0XRS8	Putative metallocarboxypeptidase ecm14	Yes	0.95	6.70	586	66.068	308810	0.7
AFUB_010920	AFUA_1G11490	B0XQR8	Endopolyphosphatase	Yes	0.78	3.40	668	76.206	295473	2.0
AFUB_052800	AFUA_5G04280	B0Y335	Uncharacterized protein	Yes	0.83	16.70	245	26.115	293027	2.3
AFUB_061470	AFUA_5G13730	B0Y269	NlpC/P60-like cell-wall peptidase, putative	Yes	0.81	12.30	359	38.763	169897	1.3
AFUB_085200	AFUA_8G01410	B0YAM7	Class V chitinase ChiB1	Yes	0.76	9.00	433	47.622	159987	1.0
AFUB_020900	AFUA_2G03830	B0XUQ5	Allergen Asp F4	Yes	0.50	25.20	322	34.093	138837	1.3
AFUB_014800	AFUA_1G15250	B0XN71	Autophagy protein Atg27, putative	Yes	0.76	8.40	333	37.377	121900	1.3
AFUB_015530	AFUA_1G16190	B0XNL0	Extracellular cell wall glucanase Crf1/allergen Asp F9	Yes	0.77	22.50	395	40.269	84105	1.0
AFUB_076030	AFUA_6G09980	B0Y842	Patched sphingolipid transporter (Ncr1), putative	Yes	0.66	4.60	1273	140.400	81393	2.0
AFUB_067180	AFUA_4G10070	B0Y6J5	Alpha-1,2-Mannosidase	Yes	0.64	1.60	641	73.606	57328	1.0

In conclusion, the results obtained with the conidial mutants were very reminiscent of the ones obtained *in vitro* with mutated recombinant RodA analyzed by ThT fluorescence assays in solution and AFM on a solid support. The rodlets of the mutants were organized differently when amino acids from both amyloidogenic regions were simultaneously mutated and/or when the protein carried a mutation that significantly extended the lag phase for rodlet formation. Although rodlets were still formed, their structural maturation required a longer time and was incomplete, leaving the conidia hydrophilic and with amorphous materials covering their surfaces.

### 2.4 Impact of point mutations on the immune response towards hydrophobins

**Fig. 8:**
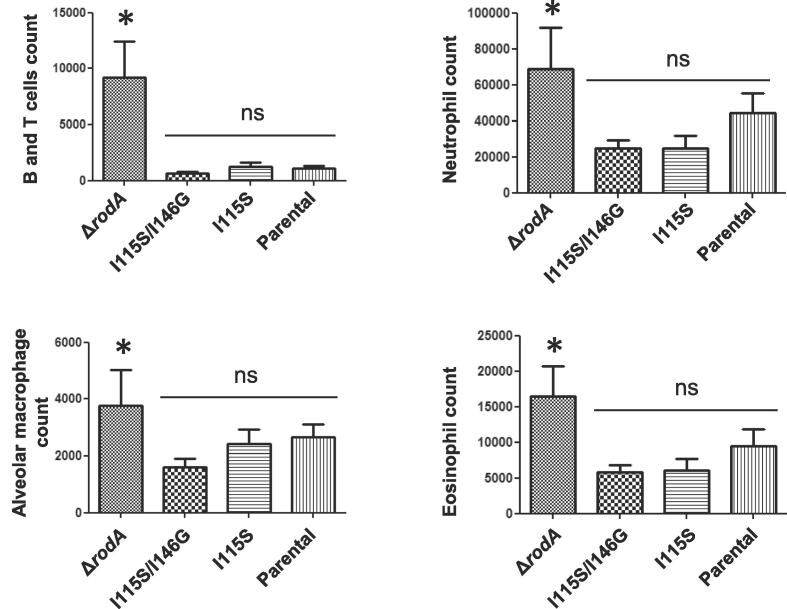
Recruitment of cells in the lungs of C57/Bl6 mice after inhalation of conidia of the single RodA I115S, double RodA I115S/I146G, Δ*rodA* mutant and the parental strain Ku80. Total number of cells were counted in the BALs (counts from >6 mice per experiment, 2 experiments) 6 h after conidial inoculation and were expressed as average with SEM. No significative differences were observed between the two mutants and the parental strain, while more cells were significantly recruited in the lungs of mice infected with the Δ*rodA* mutant.

We have reported earlier that the surface rodlet layer masks *A. fumigatus* conidial recognition by the host immune cells, whereas the conidial morphotypes devoid of a rodlet layer (i.e., Δ*rodA* mutant conidia or conidia after HF treatment) activate innate as well as adaptive immune responses (Aimanianda et al., 2009). These results prompted us to investigate if the disorganization of the rodlet structure on the conidial surface as a consequence of point mutations in the *RODA* gene would lead to the activation of immune cells by mutant conidia. Short and long term exposures of conidia from the RodA mutants and their parental strain were investigated. Intracheal inoculation of 5x10^7^ conidia into 57/Bl6 mice resulted in the immediate recruitment of cells (neutrophils, B and T cells and eosinophils), 6 h after the inoculation (Fig. 8). The most important recruitment was observed with the Δ*rodA* mutant and was similar in mice infected with the Δ*rodA* and the quadruple cysteine mutants (Fig. S11). Interestingly, the cellular recruitment with the RodA I115S/I146G mutant was similar to the parental strain, suggesting that the presence of disorganized rodlets on the conidial surface is sufficient to immunosilence the conidia. After 24 h, no difference in the GM concentration was found between all RodA mutants and the parental strain in mice lungs (data not shown). This result was in agreement with previous results (Thau et al., 1994. Valsecchi et al., 2017) indicating that rodlets are not a virulence factor for *A. fumigatus*.

**Fig. 9:**
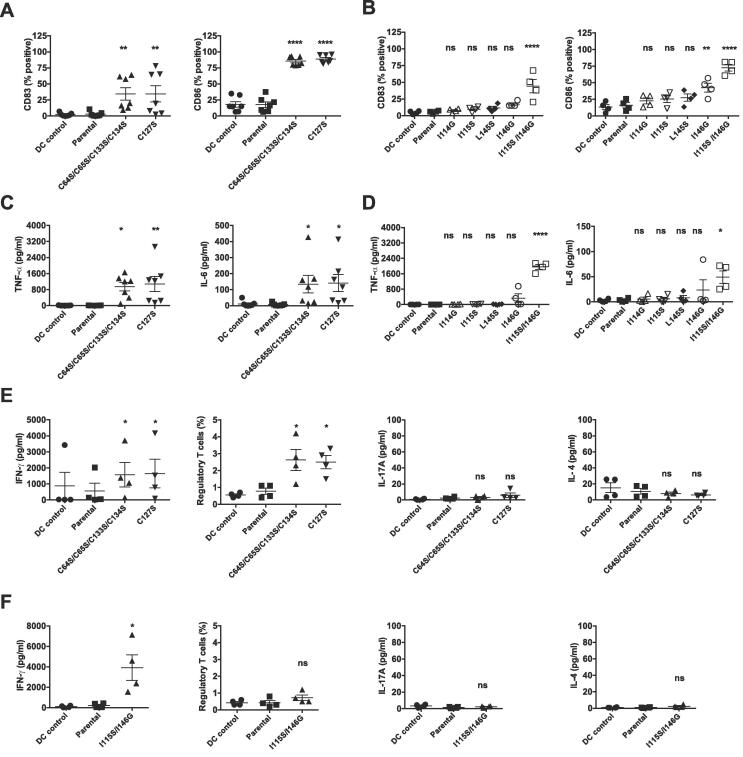
Conidia of single and quadruple cysteine mutants and the double RodA I115S/I146G mutant activate the human immune response. A-B: Induction of the dendritic cells (DC) maturation. C-D: Production of cytokines by DC. E-F: Polarization of T cell response. As other single point mutants did not alter either DC phenotype or cytokines, they were not tested for T cell response. Note that (i) the cysteine mutants also polarize the Treg response and that (ii) the I146G mutant slightly activates the DC response.

Long-term exposure was investigated with fixed conidia and human dendritic cells (DC) for 2 reasons: (i) DCs are major players in deciding the fate of the immune response and (ii) the use of fixed conidia was necessary since unfixed conidia would germinate and thus interfere with the specific immune response towards intact conidia of the parental and mutant strains. *A. fumigatus* conidia bearing mutations in the cysteine residue(s) [both single C127S or quadruple (C64S/C65S/C133S/C134S)] or the double I115S/I146G mutant conidia were indeed immunogenic in our experimental conditions. A significantly higher expression of CD83, a terminal maturation marker of DC, and of the co-stimulatory molecule CD86 was seen when DCs were exposed to cysteine (both single and quadruple) or I115S/I146G mutant conidia (Fig. 9A). Other DC maturation markers such as CD40, CD86 and HLA-DR were also enhanced by these mutant conidia (data not shown). In contrast, conidia with single mutations in the C4-C5 or C7-C8 loop regions (i.e., I114G, I115S, L145S, I146G point-mutant conidia) did not induce a DC response (Fig. 9B). Moreover, conidia with a cysteine mutation (both single or quadruple) or the double I115S/I146G mutation induced significantly higher quantities of the DC cytokines TNFα and IL6 (Fig. 9C,D). Untreated immature DCs produced only minimal quantities of cytokines like the parental or the single point mutated I114G, I115S or L145S conidia, while the I146G mutant conidia induced moderate amounts of these cytokines.

CD4^+^ T cell polarization mediated by *A. fumigatus RODA* point-mutant conidia matured DCs was also investigated. Quantification of secretion of T cell cytokines (IFN-γ, IL-4, IL-17A) or intracellular staining of the transcription factor FoxP3 revealed that cysteine mutant conidia [i.e., both C127S and C64S/C65S/C133S/C134S)] or harboring the double mutation (I115S/I146G) promoted distinct T cell responses. Single and quadruple cysteine mutant conidia-matured-DCs polarized predominantly CD4^+^CD25^+^CD127^-^ FoxP3^+^ Treg and IFN-γ (Th1) responses (Fig. 9E), whereas Th2 (IL-4) and Th17 (IL-17A) cytokines remained at basal levels (Fig. 9E). On the contrary, I115S/I146G double mutant conidia matured-DCs promoted only Th1 responses, while other T cell subsets, including Tregs, were not altered (Fig. 9F). Hence, *A. fumigatus* conidia that lack disulfide bridges in RodA or with the double mutation (I115S/I146G) in the amyloidogenic regions are both immunogenic but promoted distinct T cell responses.

**Fig. 10:**
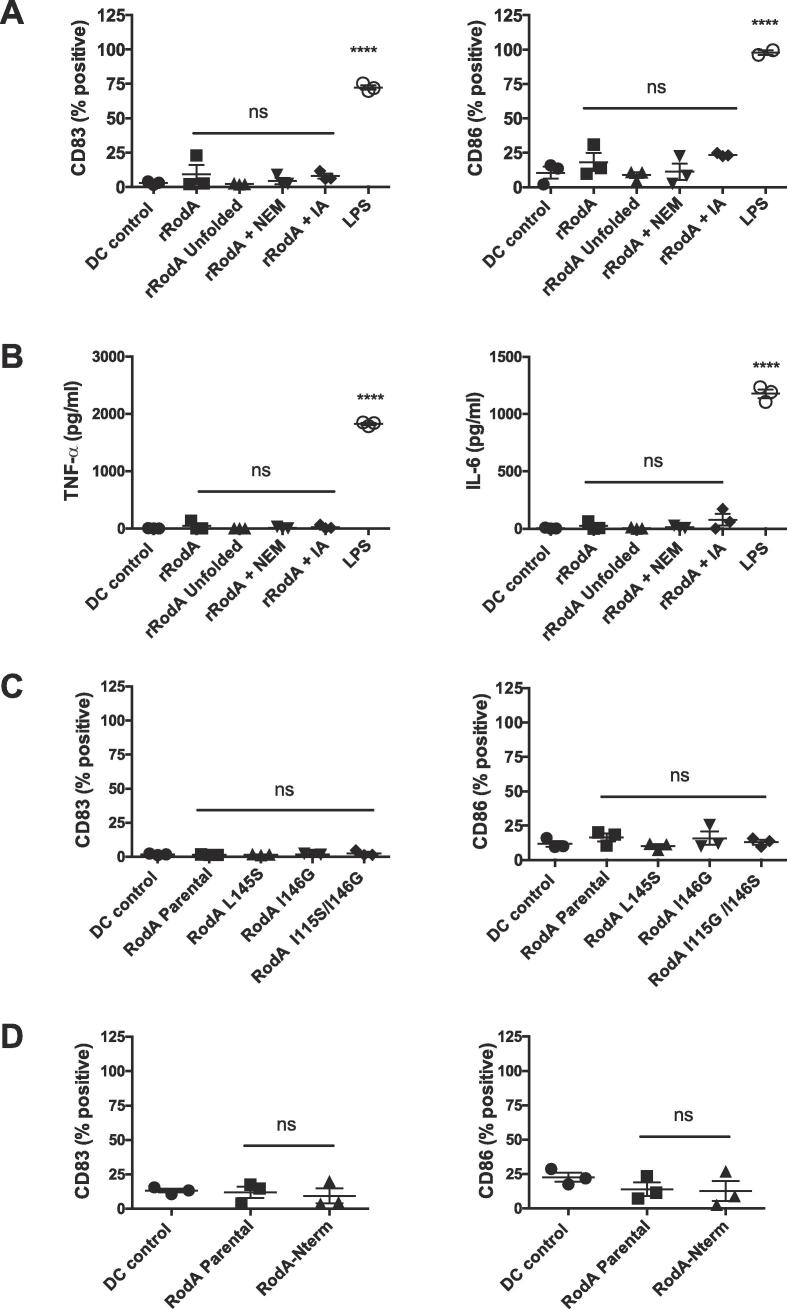
Lack of DC activation by the reduced (A,B) or mutated RodA protein (C,D). A-B: Unfolded rRodA, with or without reduced and blocked Cys residues with N-ethyl maleimide (NEM) or iodoacetamide (IA) do not activate dendritic cells (A) and do not induce cytokine production (B). C-D: RodA extracted from conidia of mutants L145S, I146G, I115G/I146S (C) or lacking the N terminal region (RodA-Nterm) (D), are immunologically inert as parental RodA. LPS is used as a control.

Earlier, we had shown that RodA extracted from the conidial surface did not stimulate an immune response of the host (Aimanianda et al., 2009). Recombinant RodA, whether folded or unfolded, with non-reduced or reduced and blocked cysteines with N-ethyl maleimide (NEM) or iodoacetamide (IA), did not modify either the DC phenotype or the production of cytokines (Fig. 10A,B). Similar results were obtained for conidia-extracted RodA harboring single or double mutations in their C4-C5 or C7-C8 regions or lacking the forty *N*-terminal amino acids (Fig. 10C,D). These observations clearly indicated that the DC activation and maturation induced by the double I115S/I146G mutant conidia was only due to the emergence of different pathogen-associated molecular patterns on the surface of conidia, concomitant with the delayed formation of the rodlets and hence with the disorganization of the outer cell wall layer.

### 2.5 Disassembly of the rodlet structure during germination

**Fig. 11:**
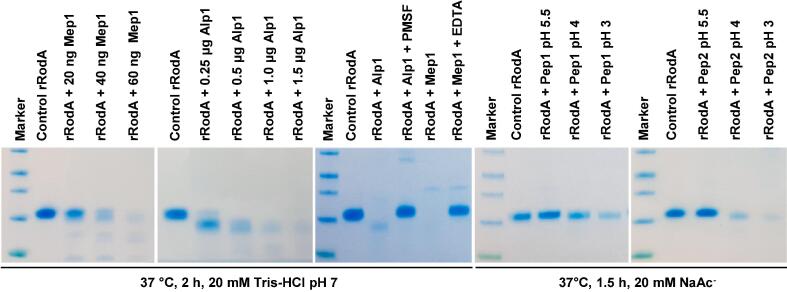
Degradation of rRodA by the major proteases identified in *A. fumigatus,* Alp1, Mep1, Pep1 and Pep2. rRodA (4 µg) was mixed with different proteases (1 µg or less as indicated) in 30 µl of 20 mM Tris-HCl pH 7 for Alp1 and Melp1 or sodium acetate pH 5.5, 4 or 3 for Pep1 and Pep2. Samples were taken after 1.5 h or 2 h of incubation at 37 °C. PMSF, EDTA or slightly acidic pH were used as inhibitors of Alp1Mep1 Pep1 and Pep2 respectively.

In nature, the activation of the immune response is associated with the loss of the rodlet layer, which occurs during conidial germination (Aimanianda et al., 2009). However, the mechanism of disintegration of the rodlet layer during germination has not been previously investigated. Disassembly of the rodlet structure is a progressive event during conidial swelling. Complete removal of the rodlet structure from the conidial surface occurs in about 5–6 h from the commencement of the germination process (data not shown).When recombinant or native RodA proteins were incubated with the ethanol precipitated protein mixture from the supernatant of germinating conidia, RodA was degraded (Fig. S12). RodA degradation was blocked by heat inactivation (data not shown), suggesting that degradation was due to proteases. Incubation of the native or recombinant RodA with the major proteases identified in *A. fumigatus* (Jaton-Ogay et al., 1994, Reichard et al., 1997, Sriranganadane et al., 2010) showed that the later proteases were able to degrade rRodA (Fig. 11). This lack of specificity and the redundancy of the proteases able to degrade rRodA explained why neither the degradation of RodA nor conidial germination were affected in the double Δalp1/Δmep1(Jaton-Ogay et al., 1994) or quadruple Δpep1/Δpep2 /ΔctsD/ΔopsB mutants that was constructed in this work (data not shown).

## 3 Discussion

### 3.1 The structure and assembly of *A. fumigatus* RodA into rodlets

Analysis of rRodA and conidial native RodA for understanding of the assembly of the rodlets has suggested that rodlet formation in *A. fumigatus* may be unusual in that the two amyloidogenic regions are likely to be involved in the formation of intermolecular β-sheet structures. In contrast in other fungi, only single amyloidogenic regions have been identified (Macindoe et al., 2012, Niu et al., 2017, Wang et al., 2004). Here, like earlier studies with the *Neurospora crassa* hydrophobin EAS (Kwan et al., 2006), we used solution NMR spectroscopy to determine the structure of the monomeric form. The RodA monomer in solution displays the β-barrel structure typical of hydrophobins and the surface of RodA exhibits a separation of charged and hydrophobic amino acid residues, which renders the protein amphipathic. Also, as for other hydrophobins, the amphiphilic nature of the RodA monomer likely plays an important role in localizing the protein at an interface and the flexibility of the amyloidogenic regions may be necessary to accommodate the structural changes required for multimerization and uniform covering of substrates. In the absence of crystals of hydrophobins in the assembled form, solid state NMR (ssNMR) has been used to probe the 3D structure of the EAS rodlets and confirmed that hydrophobin rodlets are composed of a β-sheet-rich structure with an altered protein fold compared to the monomer in solution (Morris et al., 2012). Similar ssNMR studies of RodA will likely reveal how the two amyloidogenic regions in the RodA rodlets contribute to the structure and packing of rodlets within the robust protein film.

Earlier studies have shown that an increase in pH, the presence of Ca^2+^, a low ionic strength, the polarity of the solvent, the high temperature, the presence or absence of an air:water interface, the presence of detergents, the protein concentration, specific nutriments and the physic-chemical nature of the surface are all environmental factors which can influence rodlet formation (Aimanianda et al., 2009, Cicatiello et al., 2017, Longobardi et al., 2012, Morris et al., 2011, Niu et al., 2017, Pham et al., 2016, Pham et al., 2018, Talbot et al., 1996, Talbot et al., 1993, Tanaka et al., 2017, Wang et al., 2004, Zykwinska et al., 2014a). The comparison of rodlet formation at the surface of the conidia of *A. fumigatus* and on an artificial hydrophobic surface like HOPG of wild-type or rRodA mutated proteins, has indicated that the environment influences the formation of the rodlet structure. For example, even though rRodA carrying mutations form rodlets that are similar to the wild type protein on the HOPG substrate, the rodlet structure and coverage on the conidial surface of the mutant looked altered on the more complex surface of the conidial cell wall. Similar observations have been reported for other hydrophobins like SC3 from *Schizophyllum commune* and Vmh2 from *Pleurotus ostreatus* that can also spontaneously self-assemble in aqueous solutions (Longobardi et al., 2012, Wosten et al., 1993, Zykwinska et al., 2014a). The presence of oligosaccharides facilitates the self-assembly in water of *P. ostreatus* Vmh2 and the width of the rodlet is influenced by the addition of cyclic 1,4-glucans (Armenante et al., 2010). The transition of *Schizophyllum commune* SC3 from a helical configuration to the stable β-rich amyloid conformation is promoted by the presence of soluble glucans (Scholtmeijer et al., 2009). In *A. fumigatus*, the role of the inner components of the conidial cell wall, which are located below the rodlet layer, such as α- and β-1,3-glucans, galactomannan or melanin, on the structure and anchoring of rodlets has not been investigated to date. An accurate definition of the events controlling the structural conversion of the *A. fumigatus* RodA protein into rodlets remains to be generated and should take into account the natural environment of the conidium. Moreover, the observation that there are different triggers for rodlet assembly in different hydrophobins likely reflects the fact that apart from the pattern of conserved disulfide bonds, which constrains the β-barrel core, the sequences and structures of the proteins are highly diverse (Ren et al., 2013).

*In vitro* studies, while very useful, can be limited by the homogeneous nature of the substrate and the inability to effectively probe the three-dimensional organization of the rodlet-containing layer. Moreover, the role of the cellular environment used in the heterologous host for the production of recombinant RodA protein has not been investigated. Even though *A. fumigatus* RodA protein has been produced for our study in *E. coli* and folded *in vitro* to obtain a protein that is folded like conidial RodA and is able to form rodlets, it has been previously shown that *A. fumigatus* RodA protein can be also produced in *Pichia pastoris* (Pedersen et al., 2011). The amount produced in *P. pastoris* was 200 to 300 mg/L to be compared with *E. coli* (∼2 mg/L). However, although it was verified that the recombinant RodA of *A. fumigatus* produced in *Pichia* was able to convert a hydrophilic substrate into a hydrophobic surface, the disulfide pairings, the ability of the recombinant proteins produced in *P. pastoris* to make rodlets and their glycosylation were not tested.

On conidia the rodlets are packed into distinct nanodomains, with a width of 60 to 100 nm while the height of the layer is estimated to be around 5 nm [this study and (Zykwinska et al., 2014b)], suggesting that the nanodomains are organized in multilayers on the surface of the conidium. The parallel organization of rodlets could arise from the assembly of the oligomers directly on the conidium surface or from the adhesion of rodlets aggregated in the medium prior to binding to the cell wall. The growth of one rodlet may provide a surface that favours nucleation and elongation of an adjacent rodlet, a process of secondary nucleation that has been shown to be important in the formation of some pathogenic amyloids (Gebbink et al., 2005, Pham et al., 2016). The observation that rodlets in adjacent nanodomains are oriented at angles of up to 90° may be consistent with repulsion between the outer hydrophobic components of different layers but could also be a consequence of the termination of nucleation and/or elongation when coverage of the conidium surface by rodlets removes the available surface. Interestingly, eventhough acid extraction leads to full-length RodA and a truncated version of the protein lacking 40 aminoacids (Fig. S7), incubation with hydrofluoric or formic acid did not degrade recombinant RodA into a truncated form lacking the *N*-terminal 40-amino acids. This fragment of RodA was only induced *in vitro* by the incubation of rRodA with the serine protease Alp1. These results indicate that aminoacids 40 or 41 are a sensitive exposed point in the rodlets that may be accessible to proteases from the inner cell wall.

While RodA clearly contributes to conidial hydrophobicity, an additional element to take into account is the role of other members of the hydrophobin family in controlling the hydrophobic properties of the conidium. In *A. nidulans*, all six hydrophobins contributed to hydrophobicity of the spore surface and were able to self-assemble (Grünbacher et al., 2014). However, only the deletion of *RODA* led to a mutant without rodlet on the surface even though DewA was able to form rodlets *in vitro*. When DewA and DewB were placed under the control of the *RODA* promoter in a Δ*rodA* mutant, both hydrophobins were able to produce rodlets, albeit with a different structure than the classical parental strain. Similarly, when the hydrophobins of *S. commune* (SC1 and 4) or RodA and Dew A of *A. nidulans*, or EAS of *N. crassa* were expressed from the *MPG1* promoter of *M. oryzae*, they were also able to complement, although only partially, the *MPG1* mutation, showing at the same time the lack of specificity of the hydrophobin aggregation but the need to recognize specific fungal features to induce the proper formation of rodlets on the conidial surface (Scholtmeijer et al., 2009, Kershaw et al., 1998). In *Beauvaria bassiana*, the single hydrophobin Hyd1 is able to form rodlets although the rodlets are truncated and incomplete suggesting that the two hydrophobins Hyd1 and 2 of this species act together to form the rodlets. In contrast in *A. fumigatus*, although eight hydrophobin genes (*RODA* to *G*) were identified (Jensen et al., 2010), only RodA was responsible for the formation of rodlets since a septuple *RODB/C/D/E/F/G* mutant with all these genes deleted but with wild-type RodA appeared like the parental strain (Valsecchi et al., 2017). The function of the other hydrophobins in *A. fumigatus* remains unknown.

### 3.2 Role of rodlets in *A. fumigatus* pathogenicity

Several biological facts suggest a putative role of rodlets in the pathogenicity of *A. fumigatus,* although pros and cons arguments have been published. First, the RodA protein itself, even in drastically different conformations or with point mutations is immunologically inert. The reasons for this lack of immune activation remain unknown and unlike other functional and disease-associated β-amyloids, which stimulate the innate cells via TLR and inflammasome activation (Halle et al., 2008, Tükel et al., 2009), the structure of of RodA does not dictate immune inertness. Our data on RodA and recent unpublished data (Heddergott and Latgé) on other cysteine-rich proteins of low Mr suggests that the amino acid sequence is responsible for the lack of immunogenicity against RodA. Second, by masking inner cell wall PAMPs like β-glucans and mannans, the rodlets are responsible for the lack of immediate recognition of conidia by the immune cell pattern recognition receptors (Bayry et al., 2014, Beauvais et al., 2013). In a murine model of corneal infection, the Δ*rodA* mutant is less pathogenic because of an increase in cytokine production, which boosts the anti-fungal immune response (Carrion et al., 2013). However, in our current animal experiment with intra tracheal inoculation of C57/Bl6 mice or in earlier assays with OF1 using intranasal inoculations and severe corticosteroid/cyclophospahmide immunosuppressions (Thau et al., 1994, Valsecchi et al., 2017), no difference in pathogenicity of the RodA mutants and parental strain was observed. Third, conidial rodlets have a negative impact on the innate immune mechanisms: they reduce NET formation when neutrophils come into contact with conidia and the addition of RodA to Δ*rodA* mutant conidia reduces the formation of NET (Bruns et al., 2010) and decrease macrophage efficacy (Dagenais et al., 2010). Fourth, the rodlet layer makes the conidium less permeable to the external antifungal molecules. The loss of rodlets and change in hydrophobicity that occurs during germination is indeed associated with an increased sensitivity to antifungal drugs (Dague et al., 2008a). Finally, hydrophobins favour the binding of enzymes to their substrate and stimulate their enzymatic activity (Pham et al., 2016, Ribitsch et al., 2015, Takahashi et al., 2005, Tanaka et al., 2017). This has been especially shown with MPG1 and two orthologs of RodA, namely *A. oryzae* RolA and *A. nidulans* RodA that have been shown to interact via ionic interaction with enzymes such as cutinases. It remains to be investigated whether fungal enzymes involved in pulmonary matrix degradation and host collectins and antimicrobial peptides bind to hydrophobin rodlets and play a role during lung infection by *A. fumigatus*.

## 4 Conclusions

The hydrophobin, RodA, forms functional amyloids with a rodlet morphology that coat *A. fumigatus* conidia. Assembly of parental and point mutated RodA proteins *in vitro* and on the conidial surface was analyzed with multiple technical approaches. Proper rodlet secretion and assembly requires the presence of intact cysteine bridges and two amyloidogenic regions. Even when disorganized, the rodlet layer is able to immunosilence the conidium. Complete degradation of the rodlet layer during germination by cell wall proteases activates immune cells. These studies have shown that rodlets are a dynamic structure that continuously evolves from conidial formation to germination and play a role during the early establishment of aspergillosis. Progress in understanding the mechanisms of self-assembly and the rodlet structure may also lead to advances towards solving medical problems associated with amyloid aggregation.

## 5 Material and methods

### 5.1 Extraction of the native RodA from conidia of *A. fumigatus*

RodA was extracted from dry conidia of *A. fumigatus* by a 2 h incubation on ice with formic acid. The material extracted was similar to the one extracted with a 48% hydrofluoric acid extraction for 72 h at 4 °C shown before to isolate rodlets from conidia (Aimanianda et al., 2009). The presence of rodlets was analyzed by SDS-PAGE using a 15% polyacrylamide gel and by Western blotting with an anti-RodA antibody (Valsecchi et al., 2017).

### 5.2 Production of the recombinant RodA protein for NMR analysis

The sequence used to prepare the recombinant RodA protein for solution NMR corresponds to residues 19-159 of *A. fumigatus* A1163 RodA (AFUB_057130) without the predicted N-terminal secretion peptide and containing an extra serine N-terminal residue that arises from cloning. The protein was obtained in lyophilized form as described (Pille et al., 2015) with a slight modification. Briefly, the HisTag-ubiquitin-rRodA fusion protein was encoded on a pET-28b(+) plasmid (Proteogenix) conserving the C-terminal ubiquitin GG recognition motif for deubiquitinases*.* The proteolysis step to separate RodA from the *hexa*-histidine-tagged ubiquitin was performed with His-tagged-deubiquitinase UBP41. Uniformly ^15^N and ^15^N/^13^C doubly labeled rRodA was expressed in minimal media containing ^15^NH_4_Cl and U-^13^C_6_ glucose (Euriso-top, Saclay) as sole sources of nitrogen and carbon, respectively. The rRodA was obtained after *in vitro* oxidative refolding. Integrity, label and sequence of the protein were analyzed by mass spectrometry. Protein samples were typically prepared at 0.36–0.69 mM concentrations in 20 mM CD_3_COONa pH 4.3 10% D_2_O. No dependence of line width or chemical shifts, and no signal intensity variation over time were observed in ^1^H-^15^N HSQC between 0.10 and 0.69 mM concentrations, indicating that the soluble form does not self-assemble in this concentration range and that samples were stable.

Doubly labeled conidial RodA was extracted from *A. fumigatus* conidia grown in minimal media supplemented with ^15^NH_4_Cl and U-^13^C_6_ glucose. Mature conidia were harvested and washed extensively in Tween 20 0.05%. To recover RodA protein, lyophilized conidia were incubated with 100% formic acid on ice (10 min to 2 h). After removal of the conidia by centrifugation, the supernatant was flushed with gaseous nitrogen to remove formic acid and the protein was then dissolved in water. NMR samples were prepared at 50–60 µM concentrations in the same buffer that was used for recombinant RodA.

### 5.3 NMR and structure calculation methods

Experiments were performed on a 14.7 T Direct Drive 600 NMR System spectrometer (Agilent Technologies, Santa Clara) equipped with a triple resonance cryogenic probe. Spectra were recorded at 25 °C using VnmrJ 3.2A (Agilent). Processing and analysis were performed with NMRPipe and CCPNMR Analysis, respectively. Proton, carbon and nitrogen resonances were assigned to backbone and side-chains atoms following a standard strategy with 2- (2D) and 3-dimensional (3D) heteronuclear experiments as described by Pille and co-workers (Pille et al., 2015). Assignment of RodA extracted from conidia was performed by comparing ^1^H-^15^N and ^1^H–^13^C HSQC, HNCO, HNCA experiments with the corresponding spectra of rRodA. Backbone amide ^15^N heteronuclear ^1^H-^15^N nOe (nuclear Overhauser enhancement) spectra were recorded to analyze the internal dynamics of rRodA, with a saturation/no-saturation delay of 3 s and a total recovery delay of 3.07 s. To obtain distance constraints, 3D ^15^N and ^13^C (aliphatic and aromatic regions) edited HSQC-nOesy were recorded with a 120 ms mixing time for nOe build-up (Kay et al., 1993, Muhandiram and Kay, 1994). Dihedral Phi and Psi angle constraints were calculated with Talos-N (Shen et al., 2009). An HNHA spectrum was used to validate Talos-N Phi angle constraints. Assignment of nOes and structure calculations were performed using ARIA 2.3.2 (Rieping et al., 2007) coupled to CNS 1.2.1 (Brunger et al., 1998). ARIA was run with a log-harmonic potential and automatic weighting of constraints spin diffusion correction, network anchoring and explicit water refinement using standard protocols. The ten lowest total-energy conformers were selected out of the 500 structures calculated in the final run. Structures were calculated with the disulfide bridge pairing determined experimentally for native EAS (Kwan et al., 2006) and observed in other recombinant hydrophobins [C1-C5 (56–133), C2-C6 (64–127), C3-C4 (65–105), C7-C8 (134–152)]. Herein, the cysteines are noted by their order of appearance in the sequence (C1 to C8) or by their sequence position. A careful analysis of the topology of the molecule (antiparallel β-sheets involving cysteines), of the network of nOes supporting the topology and the unambiguous assignment of several nOes between cysteines and involving neighbouring residues clearly demonstrated that rRodA showed the topology determined for native EAS. Structures were visualized and analyzed with PYMOL (Schrödinger LLC). Secondary structure content of RodA was determined according to DSSP. The quality of the structures was assessed with PROCHECK 3.5.4 (Laskowski et al., 1993), WHATCHECK (Hooft et al., 1996), MOLPROBITY (Chen et al., 2010) and PROSA (Sippl, 1993). The coordinates have been deposited to the pdb under the accession code 6GCJ.

### 5.4 Production of the rRodA and mutant proteins for Thioflavin T (ThT) fluorescence and AFM imaging

Production of rRodA proteins, wild type and mutants, was achieved as previously described for the hydrophobin MPG1(Pham et al., 2016) apart from the following three changes. Fusion proteins were expressed using a modified version of the pHUE plasmid, encoding a version of ubiquitin with the C-terminal GG residues of ubiquitin replaced by a TEV protease recognition sequence. Oxidative refolding was achieved by dialysis against 10 mM reduced glutathione, 1 mM oxidized glutathione, 50 mM sodium acetate, 100 mM sodium chloride, pH 5.0) with two changes of redox buffer (6 h each at 4 °C; protein:buffer 1:25 (v/v)). Cleavage of hydrophobin from His_6_-ubiquitin occurred by addition of TEV enzyme. After reverse phase HPLC, the correct folding of rRodA and variants was confirmed by 1D ^1^H nuclear magnetic resonance spectroscopy.

### 5.5 Thioflavin T (ThT) rodlet self-assembly assay

Lyophilized rRodA and mutant proteins were first solubilized in deionized H_2_O. The stock protein solutions were diluted in ThT buffer (40 µM Thioflavin T in 20 mM sodium acetate, pH 5.0) to a final protein concentration of 25 µg/mL (1.73 µM). Samples were prepared to 100 µl in a black, clear-bottom 96-well microplate (Greiner Bio-One). The microplate was incubated at 50 °C and fluorescence recorded with 440/480 nm excitation/emission filters following every 90 s of double orbital shaking at 700 rpm in a BMG Labtech POLARstar Omega multi-mode microplate reader BMG Labtech Australia, VIC, Australia. All protein samples in each independent experiment were analyzed in triplicate. Each sample triplicate was normalized and graphed individually. Lag phase and time to reach half of the maximum fluorescence (t_1/2_) was interpreted from mean of triplicates. Lag phase and t_1/2_ were determined by the last time point before the normalized ThT fluorescence reached a value of 0.1 and the time at which normalized 0.5 ThT fluorescence reached 0.5, respectively. Statistical analysis was performed on GraphPad Prism (version 6.04 for Windows, GraphPad Software, CA, USA, www.graphpad.com). Data was evaluated using one-way ANOVA followed by Tukey's multiple comparisons test.

### 5.6 Surface structure of *in vitro* produced recombinant proteins and peptides seen with atomic force microscopy or transmission electron microscopy

Protein samples were diluted to 5 µg/mL (0.35 µM) in deionized H_2_O. For rRodA-WT and point mutations, a 50 µl drop of protein was deposited onto a freshly-cleaved highly-ordered pyrolytic graphite (HOPG) surface (Holgate Scientific, NSW, Australia) and allowed to dry down in a covered, ambient chamber overnight at room temperature. rRodA mutant proteins containing multiple mutations were prepared similarly, but were incubated at 50 °C in a humid environment for 2 h, followed by overnight drying in the ambient chamber. The HOPG surfaces were then further dried in a 70 °C oven for 2 h to remove any excess moisture.

The morphology of the assembled protein layers on the HOPG surface was imaged at ambient temperature using a Multimode Nanoscope® III atomic force microscopy (Veeco, CA, USA). The HOPG surfaces were scanned with tapping mode using a silicon scanning probe with tip radius <10 nm, force constant of 40 N/m and resonance frequency of 300 kHz (Tap300AI-G, BudgetSensors™, Bulgaria). The images were further processed with Gwyddion software (version 2.38, http://gwyddion.net).

For TEM, a 20-µL droplet of peptide (200 µg/mL) was placed on a formvar/carbon coated copper TEM grid (200-mesh, ProSciTech), and incubated for 2 min at room temperature. Excess solution was removed with filter paper and grids were washed twice by floating the grids on water droplets and wicking away the excess solution. Samples were stained with 2% uranyl acetate and imaged on a JEOL 1400 JEOL TEM equipped with a Morada G3 16 megapixel side camera. All images were analyzed with ImageJ.

### 5.7 Construction of the RodA point mutated and N-terminus minus ΔrodA mutants

#### 5.7.1 Construction of the template plasmid for mutagenesis

The template for directed mutagenesis reactions was constructed with the pUC19 plasmid containing the RodA upstream region (1486 bp), the RodA open reading frame (583 bp), the hygromycin cassette beta recombinase (4767 bp) (Hartmann et al., 2010) and the RodA downstream region (853 bp). The pUC19 plasmid was digested with *Sac*I and *Pst*I and the hygromycin cassette was excised with *Fsp*I from plasmid pSK529 (Hartmann et al., 2010) and the corresponding band was purified from an agarose-gel. The RodA upstream region, open reading frame and downstream region were amplified by PCR using as template the gDNA of the CEA17_Δ*akuB*^KU80^ strain (da Silva Ferreira et al., 2006) and primers 1, 2, 3, 4 (see Supplementary Table 3). The final template plasmid was assembled with the GeneArt Seamless Cloning and Assembly kit (Thermo Fisher).

#### 5.7.2 Construction of the point mutated RodA and N terminus minus strains

RodA point mutants were obtained using the QuikChange Multi Site-Directed Mutagenesis Kit (Agilent technologies) following the manufacturer instructions. Each PCR-mutagenesis reaction was performed with the template plasmid described above and primers as described in Supplementary Table 3. PCR-mutagenesis reactions for mutations C127S, C64S/C65S/C133S/C134S, I114G, I115S, L145S, I146G and D140G were performed with the template plasmid described above and primers 5, 6–7, 8, 9, 10, 11 and 12 (Supplementary Table 3), respectively. The plasmid for the double mutation I115S/I146G was generated with the RodA I146G plasmid and primer 9. DNA sequencing was performed after each mutagenesis reaction to verify the presence of the expected mutation. Each mutated plasmid was digested with the *Sph*I restriction enzyme before electroporation on the CEA17_Δ*akuB*^KU80^ parental strain as described by Lambou *et al*. (2006). Transformants were selected with 150 µg/ml of hygromycin. PCR and Southern blot analysis were performed to verify the transformants. In the Southern blot analysis, the gDNAs of mutated strains were digested with *BstZ*17I and annealed with a PCR-DIG labeled probe (Roche) of the RodA ORF (Fig. S5). A PCR was performed using primers that anneal outside the integration site of the cassette for DNA sequencing in order to verify the frame, the point mutations and the presence of the 120 bp remaining after the removal of the *HPH^R^-βrec* cassette.

The RodA-N terminal cassette was constructed with four DNA fragments, I, II, III, IV described in Fig. S4. Fragments I, II and IV were amplified by PCR with the following pairs of primers 1–13, 14–2 and 3–4 (see Supplementary Table 3). Fragment I and II contain the *RODA* upstream region and the *RODA* ORF except the DNA bases from C55 to C112 corresponding to the aminoacids P20-V39. Fragment III, corresponding to the HRP^R^-βrec resistance, was digested with *Fsp*I from plasmid pSK485followed by gel band purification. Fragment IV contains the *RODA* downstream region. The 4 fragments were assembled each other by GeneArt Seamless Cloning and Assembly kit (Thermo Fisher) and were used for electroporation after digestion with *Sph*I, on the CEA17 Δ*aku*B^KU80^ parental strain (da Silva Ferreira et al, 2006).

#### 5.7.3 Complementation of RodA I146G and C64S/C65S/C133S/C134S strains

The same plasmid constructed as a template for the mutagenesis reaction was used to complement the RodA I146G and C64S/C65S/C133S/C134S strains. This plasmid was digested with SphI before transformation by electroporation (Lambou et al., 2010).

#### 5.7.4 Growth conditions of the *A. fumigatus* strains and analysis of the conidial phenotype of the mutants

All the strains were maintained on 2% malt agar slants at room temperature. Conidia were recovered from these slants using 0.05% Tween20 aqueous mixtures and conidial germination was followed microscopically and quantified on 2% Malt agar medium. Survival of the conidia in presence of SDS (0.01 to 0.1%) and H_2_O_2_ (0.5 to 3 mM) was also tested to evaluate cell wall permeability changes.

### 5.8 Atomic force microscopy of conidia

Conidial surfaces were analyzed by AFM, using a Multimode VIII AFM (Bruker, Santa Barbara, CA). *A. fumigatus* conidia were immobilized by mechanically trapping them into porous polycarbonate membranes with a pore size similar to the conidium diameter (it4ip SA, Belgium). After filtering a concentrated suspension of conidia, the filter was rinsed with deionized water, carefully cut and attached to a metallic puck using double-sided sticky tape. The mounted sample was then transferred to the AFM liquid cell. Images were performed in contact mode under minimal applied force, using oxide-sharpened microfabricated Si_3_N_4_ tips (MSCT, Bruker) with a nominal spring constant of 0.01 N/m. The surface coverage of the amorphous layer and the rodlets were determined visually using ImageJ software.

### 5.9 Analysis of the immune response against recombinant RodA proteins and conidia of the RodA mutants

#### 5.9.1 Recombinant proteins used for studying the immune response

During the reverse phase chromatography purification step of rRodA, two main peaks corresponding to RodA are observed. The peak with lower retention time upon elution with a gradient of increasing acetonitrile in 10% methanol 0.01 %TFA) corresponded to the folded protein as determined by proton 1D and ^1^H-^15^N HSQC NMR spectra. The protein eluting with a higher retention time, either alone or with the reducing agent TCEP (Tris(2-carboxyethyl)phosphine, 5 mM) showed the characteristic NMR spectra of an unfolded protein. To produce reduced and cysteine blocked RodA samples, lyophilized, unfolded rRodA was dissolved in 6 M guanidinium hydrochloride-Tris-HCl 100 mM pH 7 or 8. Potential disulfides were reduced by incubating rRodA with DTT (dithiothreitol) at 1:10 cysteine/DTT ratio at 37 °C under a nitrogen flow. Reduced cysteines were then alkylated by adding a ten-fold molar excess of alkylating agent relative to DTT at pH 7 with iodoacetamide or at pH 8 with N-ethyl maleimide and incubating the sample 1 h at 37 °C under a nitrogen flow. Alkylation was stopped with a ten-fold molar excess of β-mercaptoethanol relative to the alkylating agent. Alkylated rRodA samples were purified by reverse phase chromatography like rRodA, lyophilized and kept at 4 °C. SELDI (surface enhanced laser desorption ionization time of flight) mass spectrometry indicated a complete labelling for IA, and two species with 7 or 8 alkylated cysteines for NEM.

Recombinant RodA wild type and proteins carrying mutations were produced as described above. Conidia from parental and mutant strains were produced after 3, 10 or 30 days of culture on 2% malt agar. Conidia were recovered from slants by vortexing the culture with an aqueous 0.05% Tween 20 solution. Conidia were extensively washed with water. Surface material of the mutant conidia was extracted after incubation of the conidia for 2 h in formic acid. The material was dialyzed before being tested immunologically.

#### 5.9.2 Human material for immune analysis

PE-conjugated MAbs to CD83 (clone HB15e), CD127 (clone HIL-7R-M21); FITC-conjugated MAbs to CD86 (clone 2331 (FUN-1)), CD25 (clone M-A251); Alexa 700-conjugated MAbs to CD4 (clone RPA-T4) were from BD Biosciencies. APC-conjugated MAbs to FoxP3 (clone 236A/E7) and Fixable Viability Dye eFluor 506 were from eBioscience.

Peripheral blood mononuclear cells (PBMCs) were obtained from buffy bags of healthy donors by Ficoll density gradient centrifugation. Anonymized buffy bags of the healthy blood donors were purchased from Centre Necker-Cabanel, Etablissement Français du Sang, Paris, France. Ethical committee permission was obtained for the use of buffy bags of healthy donors (Institut National de la Santé et de la Recherche-EFS ethical committee convention 15/EFS/012). Monocytes were isolated from PBMCs by positive selection using CD14 MicroBeads. Monocytes (0.5 × 10^6^ cells per ml) were cultured in the presence of granulocyte–macrophage colony-stimulating factor (GM-CSF; 1,000 IU per 10^6^ cells) and IL-4 (500 IU per 10^6^ cells) (both cytokines from Miltenyi Biotec) for 5 days to obtain immature monocyte-derived DCs.

Autologous naïve T cells were negatively isolated in a two-step process. First untouched CD4 T cells were isolated using the CD4 T cell isolation kit II. In the second step CD4 T cells were labeled with CD45RO and CD25 and the negative fraction containing the CD4^+^CD45RA^+^CD25^−^ was used for assessing CD4^+^ T cell polarization. All the isolation kits were purchased from Miltenyi Biotec (Paris, France).

#### 5.9.3 Treatment of DCs with RodA proteins and conidia

Immature DCs (0.5x10^6^/ml) were cultured in the presence of GM-CSF and IL-4 alone or with parental or mutant conidia (1:1) for 48 h. In other experiments, DCs were incubated with p-formaldehyde fixed conidia or various forms of RodA (1 μg/0.5 × 10^6^ DCs/ml) or LPS (100 ng/0.5 × 10^6^ DCs/ml) as described in Aimanianda et al, 2009. After 48 h of incubation, cell-free culture supernatants were collected for analysis of IL-6 and TNFα DC-cytokines (ELISA Ready-SET-Go, eBioscience). DCs were processed for flow cytometry by surface staining with fluorochrome-conjugated antibodies. Data were acquired on a LSR II (BD Biosciences) flow cytometer and analyzed using the BD FACS DIVA software (BD Biosciences).

#### 5.9.4 DCs-naïve CD4^+^ T cell co-culture

To explore the effect of *A. fumigatus* conidia-stimulated DCs on CD4^+^ T cell polarization, after stimulation, DCs were extensively washed and co-cultured with 0.1 × 10^6^ CD4^+^CD45RA^+^CD25^-^ autologous naïve T cells at a 1:20 ratio for five days in serum-free X-VIVO medium. Cell-free supernatants were collected after 5 days. Surface staining of T cells was performed with fluorescence-conjugated mAbs against CD4, CD25 or CD127. Then, cells were fixed, permeabilized using an intracellular staining kit (eBioscience) and incubated at 4 °C with fluorochrome-conjugated mAbs against FoxP3. Data were recorded on a LSR II (BD Biosciences) flow cytometer and analyzed with the BD FACS DIVA software (BD Biosciences). The cell-free culture supernatants were collected and analyzed for various T cell cytokines by ELISA (IFN-γ, IL-4, and IL-17A; ELISA Ready-SET-Go, eBioscience).

#### 5.9.5 Mouse experiments

Mouse experiments were performed with 5–6 weeks old C57/Bl6 females. A suspension of 5x10^7^ conidia in 50 µl of PBS was intratracheally inoculated per mouse and cell recruitment was investigated 6 h after inoculation. Fungal development was followed by the quantification of galactomannan in lung digests as described earlier. The cells were identified by FACS with different markers, which were CD11c^-^ Ly6G^-^CD3^+^ for T cells, CD11c^-^ Ly6G^-^CD19^+^ for B cells, CD11c l^ow^ Ly6G^high^CD11b^high^ for neutrophils CD11c^+^ siglec F ^+^CD11b^-low^ for alveolar macrophages, CD11c^-^ siglec F ^+^CD11b^high^ for eosinophils.

### 5.10 Proteolytic degradation of RodA

Native RodA (5 µg in 30 µl of 20 mM sodium acetate buffer pH 5.5) recovered from conidia with hydrofluoric acid was incubated for 2 h with an ethanol-precipitated protein mixture from the culture supernatant (2 µg of protein) of *A. fumigatus* grown in Brian’s medium at 37 °C for 24 h. The nature of the proteases degrading RodA was investigated by addition of different protease inhibitors at 1 mM concentration. The specificity of the proteolytic degradation was investigated with recombinant species of the major proteases of *A. fumigatus*, the aspartic acid proteases rPep1, rPep2, the serine protease rAlp1 and the metalloprotease rMep1 produced as described earlier (Sarfati et al., 2006). Proteolysis mixtures were incubated at 37 °C and aliquots were taken for analysis at 1.5 and 2 h. Incubation conditions were the following: rRodA (4 µg) + protease (1 µg or less as indicated in Fig. 11) in 30 µl of 20 mM Tris-HCl pH 7 for Alp1 and Melp1 and 20 mM sodium acetate pH 5.5, 4 or 3 for Pep1 and Pep2. Degradation of RodA was monitored by SDS-PAGE (Fig. 11). PMSF (1 mM, added every 0.5 h due to its unstability at pH 7) and EDTA (4 mM) were used as inhibitors of Alp1 and Mep1 respectively, while pH 5.5 was used to inhibit the activity of aspartic acid proteases.

### 5.11 Mass spectrometry experiments

#### 5.11.1 In-solution digestion

Conidia were incubated in formic acid for 2 h as described above. Preliminary experiments showed that the same proteins were extracted by 10 min or 2 h incubation times. Dried samples were re-suspended in 100 µl 8 M urea, 100 mM Tris-HCl pH 8.5. Briefly, samples were reduced with 5 mM TCEP for 30 min and alkylated with 10 mM iodoacetamide for 30 min at room temperature in the dark. Protein samples were then incubated with 250 ng rLys-C Mass Spec Grade (Promega, Madison, WI, USA) for 5 h at 37 °C for the first digestion. Samples were then diluted to 2 M urea with 100 mM Tris HCl pH 8.5 and 500 ng sequencing grade modified trypsin (Promega, Madison, WI, USA) was added for the second digestion overnight at 37 °C. A second incubation with the same amount of trypsin (5 h at 37 °C) was performed to ensure a complete digestion. Digestion was stopped by adding formic acid -and peptides were desalted and concentrated on a Sep-Pak C_18_ SPE cartridge (Waters, Milford, MA, USA) according to manufacturer’s instructions.

#### 5.11.2 Mass spectrometry analysis

Tryptic peptides were analyzed on a Q Exactive Plus instrument (Thermo Fisher Scientific, Bremen) coupled with an EASY nLC 1000 chromatography system (Thermo Fisher Scientific). Sample was loaded on an in-house packed 50 cm nano-HPLC column (75 μm inner diameter) with C_18_ resin (1.9 μm particles, 100 Å pore size, Reprosil-Pur Basic C18-HD resin, Dr. Maisch GmbH, Ammerbuch-Entringen, Germany) and equilibrated in 98% solvent A (H_2_O, 0.1% FA) and 2% solvent B (ACN, 0.1% FA). Peptides were first eluted using a 2 to 18% gradient of solvent B during 112 min, then a 18 to 30% gradient of solvent B during 35 min, a 30 to 45% gradient of solvent B during 15 min and finally a 45 to 60% gradient of solvent B during 5 min all at a 250 nL/min flow rate. The instrument method for the Q Exactive Plus was set up in the data dependent acquisition (DDA) mode. After a survey scan in the Orbitrap (resolution 70 000), the 10 most intense precursor ions were selected for HCD fragmentation with a normalized collision energy set up to 27. Charge state screening was enabled, and precursors with unknown charge state or a charge state of 1 and >7 were excluded. Dynamic exclusion was enabled for 45 s.

#### 5.11.3 Data processing for protein identification and quantification

All data were searched using Andromeda (Cox et al., 2011) with MaxQuant software (Cox and Mann, 2008, Tyanova et al., 2016) version 1.5.3.8 against a *Neosartorya fumigata* (strain CEA10 CBS 144.89 FGSC A1163) (*Aspergillus fumigatus*) Uniprot database (Proteome ID UP000001699, 9942 entries) concatenated with mutated RodA protein, usual known mass spectrometry contaminants and reversed sequences of all entries. Andromeda searches were performed choosing trypsin as specific enzyme with a maximum number of two missed cleavages. Possible modifications included carbamidomethylation (Cys, fixed), oxidation (Met, variable) and Nter acetylation (variable). The mass tolerance in MS was set to 20 ppm for the first search then 6 ppm for the main search and 10 ppm for the MS/MS. Maximum peptide charge was set to seven and five amino acids were required as minimum peptide length. The “match between runs” feature was applied for samples having the same experimental condition with a maximal retention time window of 0.7 min. One unique peptide to the protein group was required for the protein identification. A false discovery rate (FDR) cut-off of 1% was applied at the peptide and protein levels. Reverse proteins and usual MS contaminants were removed before the analysis of the data. Quantification of each identified protein was performed by summing the intensities of its associated peptides.

#### 5.11.4 Analysis of protein intensity data

For the quantification of the proteins and comparison of conditions, proteins with at least two quantified intensity values among the replicates of the condition were selected for further analysis.

Selection of “condition-specific proteins” was made by the selection of proteins with peptides identified in a unique condition. For ranking these condition-specific proteins from the most abundant ones to the least in the condition where they are observed, we used the average of their observed iBAQ values among the replicates of the condition. The iBAQ algorithm consists to normalize the summed peptide intensities by the number of theoretically observable peptides for each protein and is a relevant method to rank the absolute abundance of different proteins within a single sample (Schwanhäusser et al., 2011). Since we were particularly interested in secretory proteins among all these condition-specific proteins, we used the SignalP algorithm to predict proteins with secretory signal peptides (Petersen et al., 2011). This algorithm was used with the default cut-off value, which optimizes the performance measured as Matthews Correlation Coefficient (MCC). The mass spectrometry proteomics data have been deposited to the ProteomeXchange Consortium via the PRIDE (Table 1) partner repository with the dataset identifier PXD008503.

### 5.12 Statistical analysis

One- or two-ways ANOVA analysis with ranking of Least Square Mean Differences with the Student's *t* test was undertaken using the JMP software (Carry, NC).

## Funding

This work was supported partially by the grant Aspergillus by Aviesan, the grant DEQ20150331722 10.13039/501100002915LATGE Equipe FRM 2015 and the French Australian FAST project FR110012. This work was additionally funded by the French ANR (HYDROPHOBIN ANR-10-BLAN-1309 and FUNHYDRO ANR-16-CE110020-01) and the Institut Pasteur PTR 529. AP was a recipient of an Université Pierre et Marie Curie (UPMC) fellowship. The NMR spectrometer was partially supported by the Région Ile de France (SESAME grant).

## Conflict of interest

The authors declare no conflict of interest.
