# Supplementary material for: Assembly and disassembly of Aspergillus fumigatus conidial rodlets
Source: Cell Surf. 2019 Mar 6;5:100023. doi: 10.1016/j.tcsw.2019.100023 (PMC7389560; doi:10.1016/j.tcsw.2019.100023)
Supplement: Supplementary Figs. S1–S12 [file mmc1.pptx]

## Slide 1
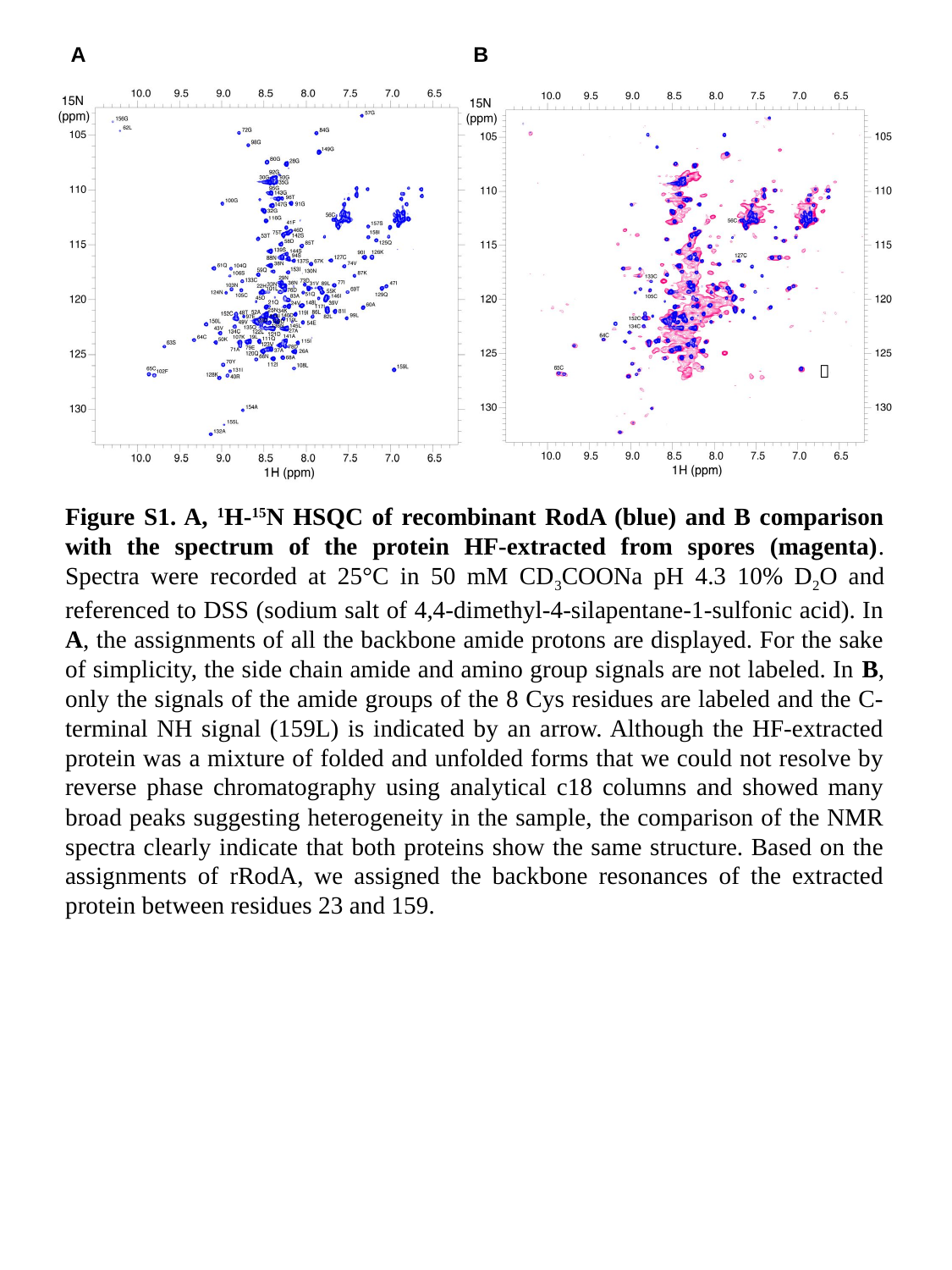

A
B

Figure S1. A, 1H-15N HSQC of recombinant RodA (blue) and B comparison with the spectrum of the protein HF-extracted from spores (magenta). Spectra were recorded at 25°C in 50 mM CD3COONa pH 4.3 10% D2O and referenced to DSS (sodium salt of 4,4-dimethyl-4-silapentane-1-sulfonic acid). In A, the assignments of all the backbone amide protons are displayed. For the sake of simplicity, the side chain amide and amino group signals are not labeled. In B, only the signals of the amide groups of the 8 Cys residues are labeled and the C-terminal NH signal (159L) is indicated by an arrow. Although the HF-extracted protein was a mixture of folded and unfolded forms that we could not resolve by reverse phase chromatography using analytical c18 columns and showed many broad peaks suggesting heterogeneity in the sample, the comparison of the NMR spectra clearly indicate that both proteins show the same structure. Based on the assignments of rRodA, we assigned the backbone resonances of the extracted protein between residues 23 and 159.

## Slide 2
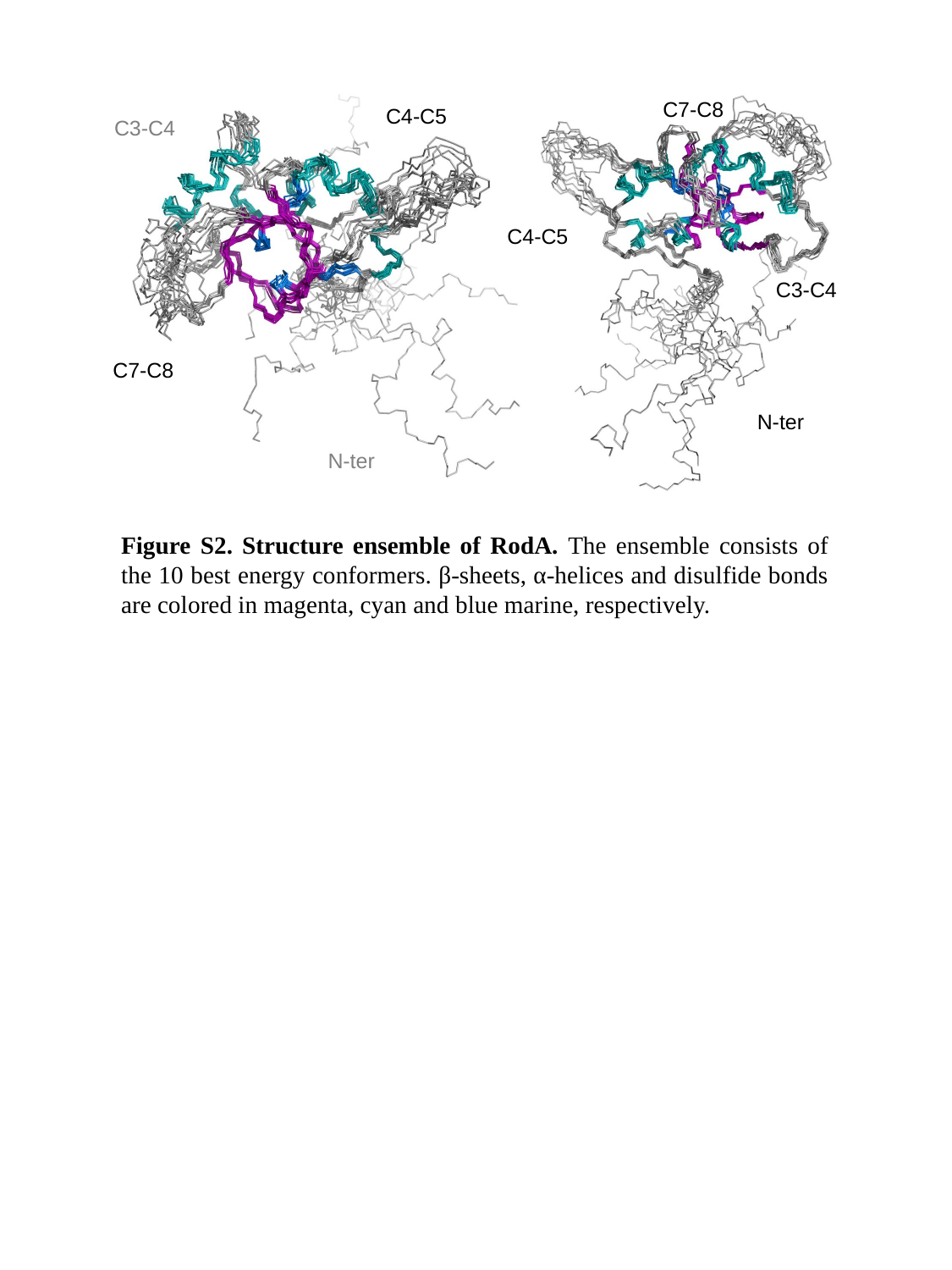

C7-C8
C4-C5
C3-C4
C4-C5
C3-C4
C7-C8
N-ter
N-ter
Figure S2. Structure ensemble of RodA. The ensemble consists of the 10 best energy conformers. β-sheets, α-helices and disulfide bonds are colored in magenta, cyan and blue marine, respectively.

## Slide 3
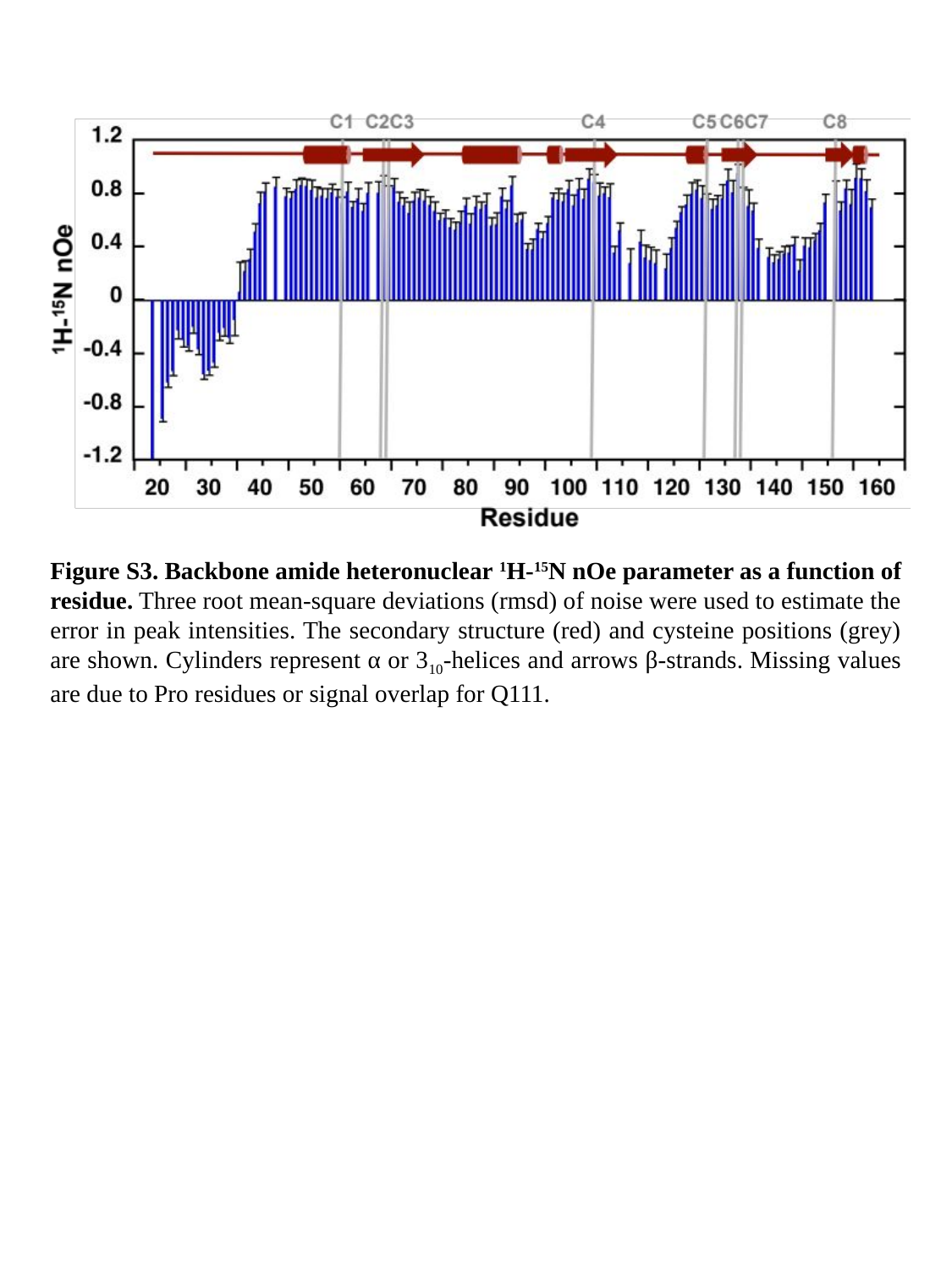

Figure S3. Backbone amide heteronuclear 1H-15N nOe parameter as a function of residue. Three root mean-square deviations (rmsd) of noise were used to estimate the error in peak intensities. The secondary structure (red) and cysteine positions (grey) are shown. Cylinders represent α or 310-helices and arrows β-strands. Missing values are due to Pro residues or signal overlap for Q111.

## Slide 4
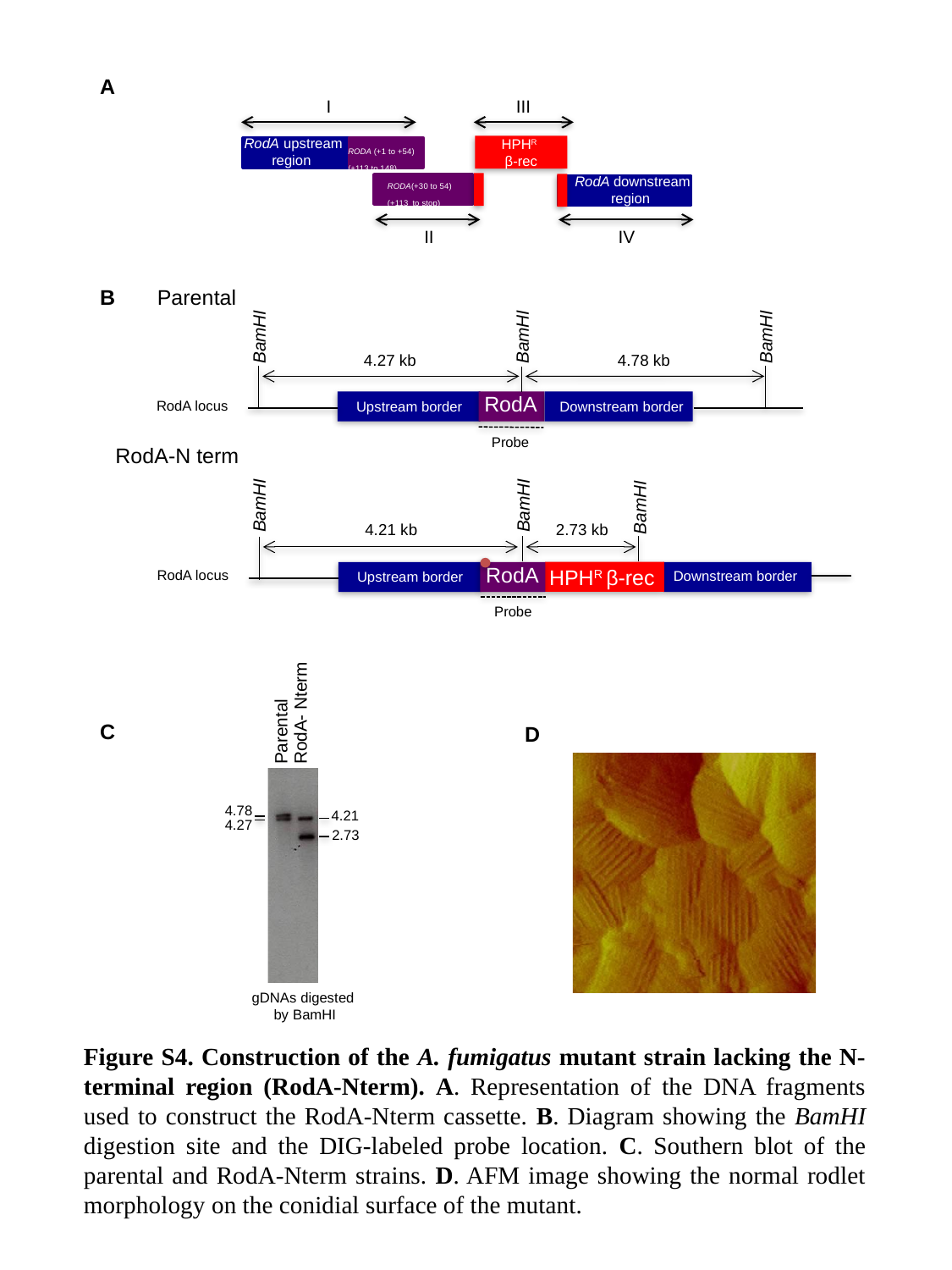

RodC
A
I
III
RodA upstream region
HPHR
β-rec
RODA (+1 to +54)(+113 to 148)
RodA downstream region
RODA(+30 to 54) (+113 to stop)
II
IV
BamHI
BamHI
BamHI
4.27 kb
4.78 kb
RodA
Upstream border
Downstream border
Probe
B
Parental
RodA locus
RodA-N term
BamHI
BamHI
BamHI
2.73 kb
4.21 kb
RodA
HPHR β-rec
Downstream border
Upstream border
Probe
RodA locus
RodA- Nterm
Parental
4.78
4.21
4.27
2.73
gDNAs digested
by BamHI
C
D
Figure S4. Construction of the A. fumigatus mutant strain lacking the N-terminal region (RodA-Nterm). A. Representation of the DNA fragments used to construct the RodA-Nterm cassette. B. Diagram showing the BamHI digestion site and the DIG-labeled probe location. C. Southern blot of the parental and RodA-Nterm strains. D. AFM image showing the normal rodlet morphology on the conidial surface of the mutant.

## Slide 5
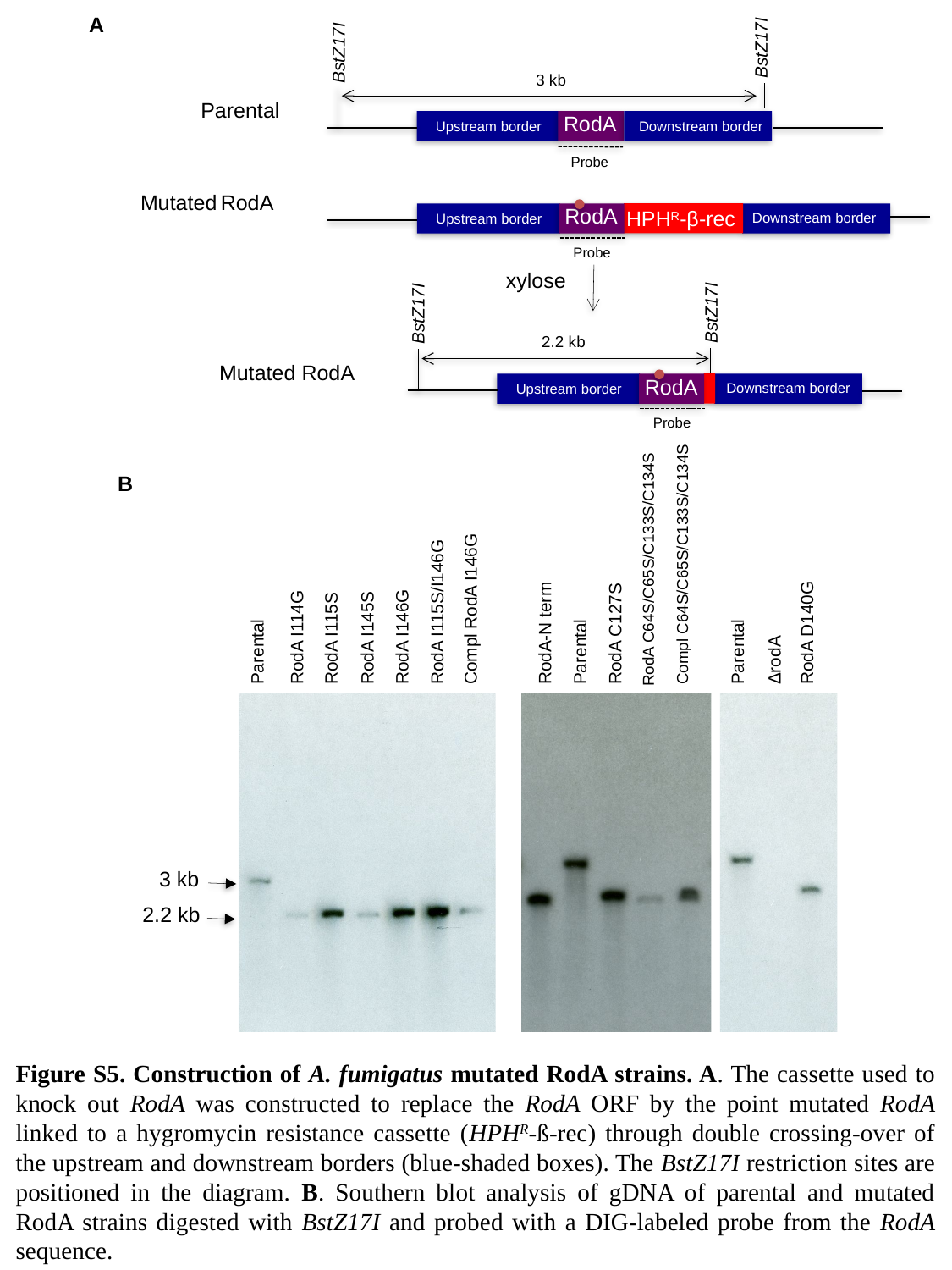

BstZ17I
BstZ17I
3 kb
RodA
Upstream border
Downstream border
Probe
Parental
A
Mutated RodA
RodA
HPHR-β-rec
Downstream border
Upstream border
Probe
BstZ17I
BstZ17I
2.2 kb
RodA
Downstream border
Upstream border
Probe
Mutated RodA
xylose
B
Compl C64S/C65S/C133S/C134S
RodA C64S/C65S/C133S/C134S
RodA I115S/I146G
Compl RodA I146G
RodA-N term
RodA D140G
RodA C127S
RodA I146G
RodA I145S
RodA I115S
RodA I114G
ΔrodA
Parental
Parental
Parental
3 kb
2.2 kb
Figure S5. Construction of A. fumigatus mutated RodA strains. A. The cassette used to knock out RodA was constructed to replace the RodA ORF by the point mutated RodA linked to a hygromycin resistance cassette (HPHR-ß-rec) through double crossing-over of the upstream and downstream borders (blue-shaded boxes). The BstZ17I restriction sites are positioned in the diagram. B. Southern blot analysis of gDNA of parental and mutated RodA strains digested with BstZ17I and probed with a DIG-labeled probe from the RodA sequence.

## Slide 6
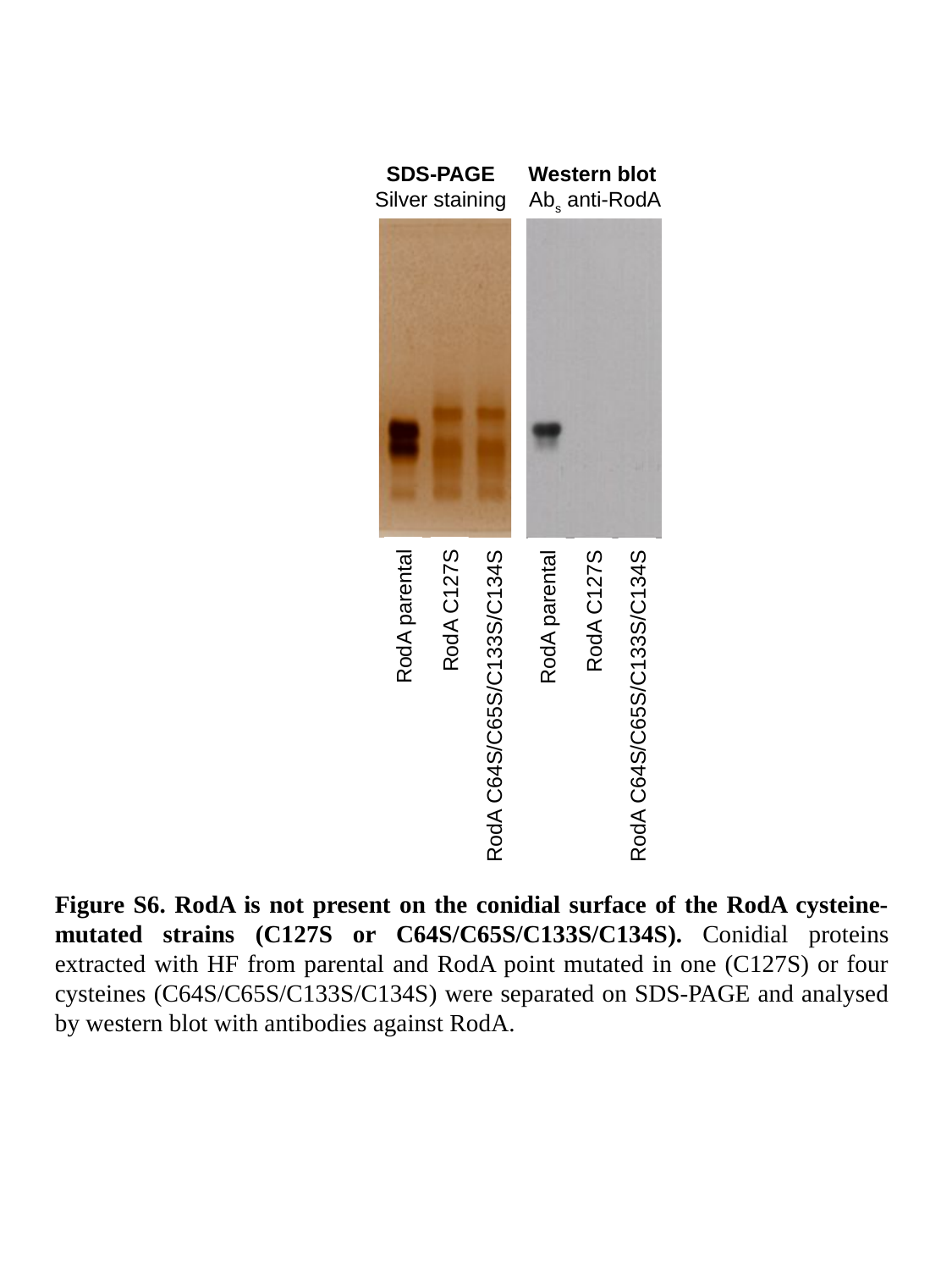

Western blot
Abs anti-RodA
SDS-PAGE
Silver staining
 RodA C127S
 RodA parental
RodA C64S/C65S/C133S/C134S
RodA C127S
RodA parental
 RodA C64S/C65S/C133S/C134S
Figure S6. RodA is not present on the conidial surface of the RodA cysteine-mutated strains (C127S or C64S/C65S/C133S/C134S). Conidial proteins extracted with HF from parental and RodA point mutated in one (C127S) or four cysteines (C64S/C65S/C133S/C134S) were separated on SDS-PAGE and analysed by western blot with antibodies against RodA.

## Slide 7
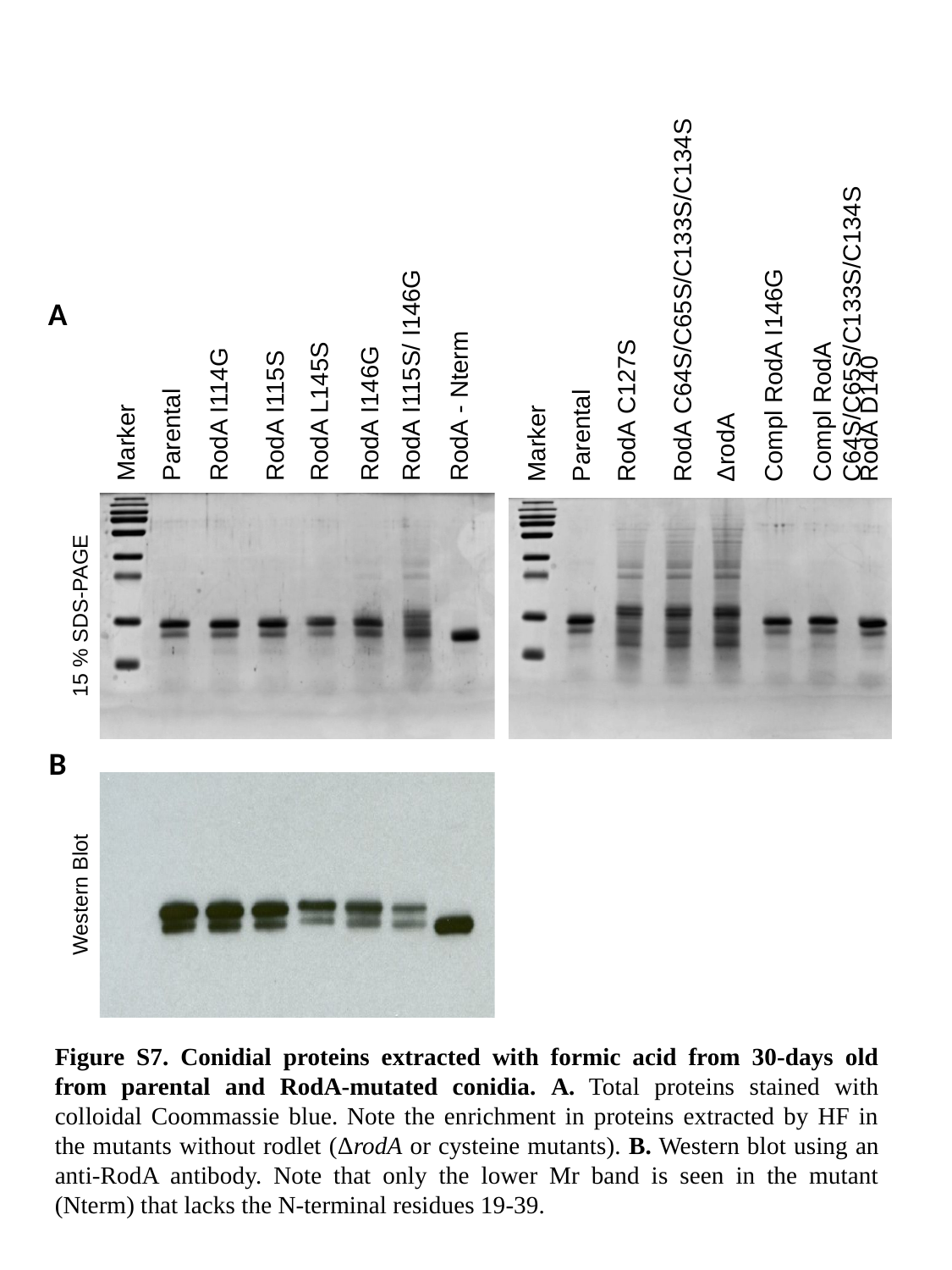

RodA - Nterm
RodA I115S/ I146G
RodA I115S
RodA L145S
RodA I114G
RodA I146G
Parental
Marker
15 % SDS-PAGE
Western Blot
Compl RodA C64S/C65S/C133S/C134S
RodA C64S/C65S/C133S/C134S
Compl RodA I146G
RodA D140
RodA C127S
ΔrodA
Marker
Parental
Figure S7. Conidial proteins extracted with formic acid from 30-days old from parental and RodA-mutated conidia. A. Total proteins stained with colloidal Coommassie blue. Note the enrichment in proteins extracted by HF in the mutants without rodlet (ΔrodA or cysteine mutants). B. Western blot using an anti-RodA antibody. Note that only the lower Mr band is seen in the mutant (Nterm) that lacks the N-terminal residues 19-39.
A
B

## Slide 8
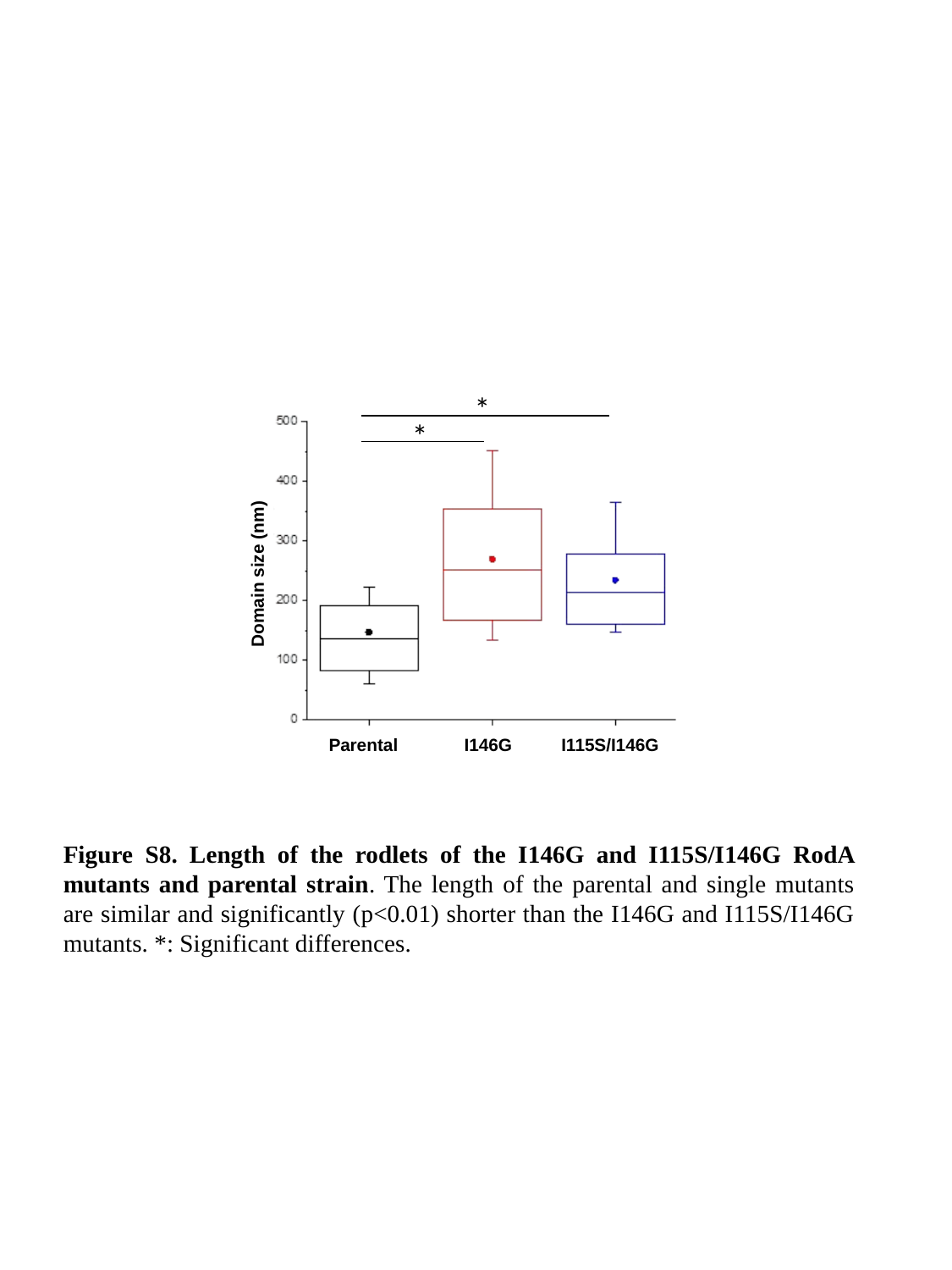

*
*
Parentall
I146Gl
I115S/I146Gl
Domain size (nm)l
Figure S8. Length of the rodlets of the I146G and I115S/I146G RodA mutants and parental strain. The length of the parental and single mutants are similar and significantly (p<0.01) shorter than the I146G and I115S/I146G mutants. *: Significant differences.

## Slide 9
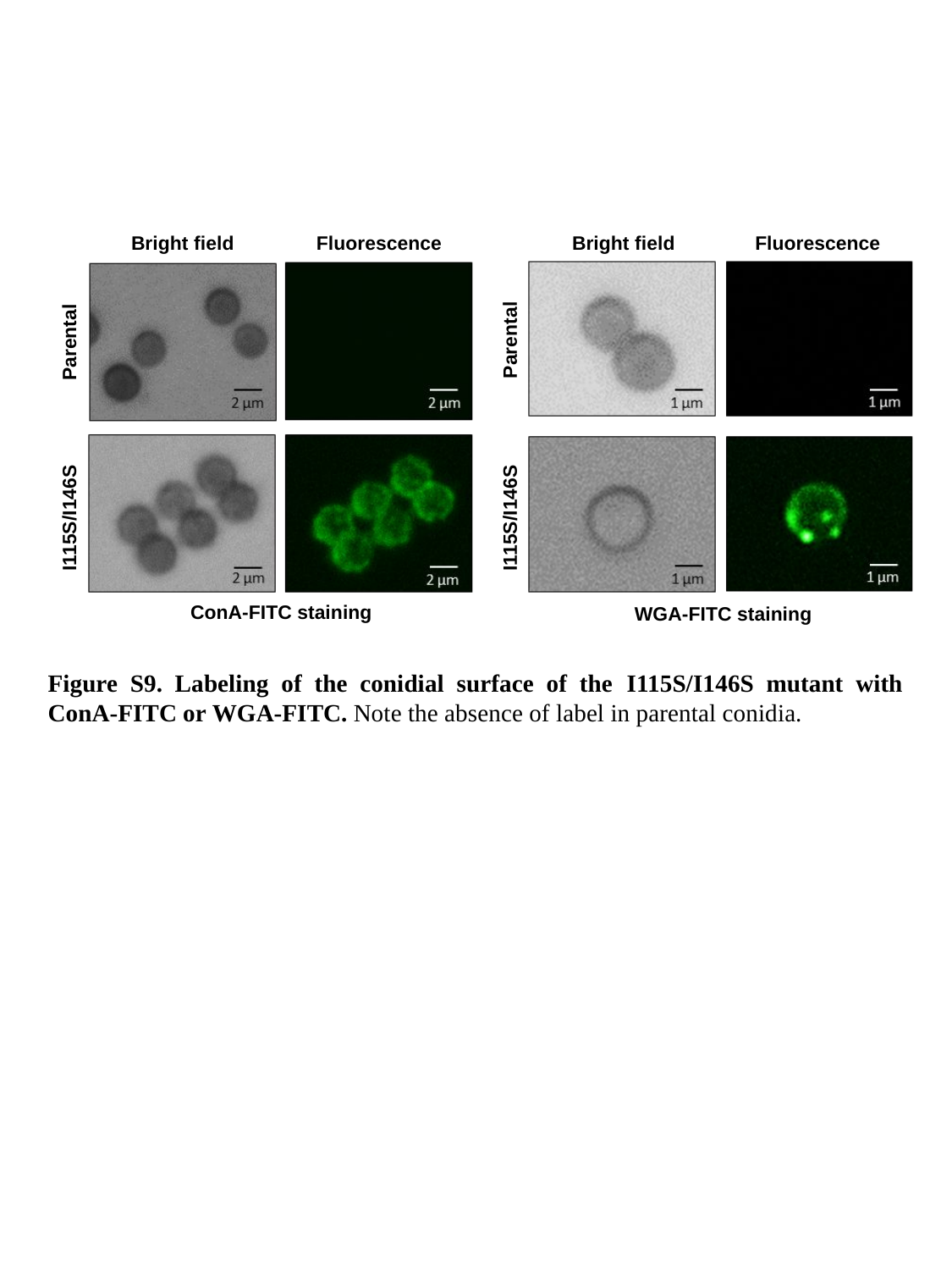

Bright field
Fluorescence
Bright field
Fluorescence
Parental
Parental
I115S/I146S
I115S/I146S
ConA-FITC staining
WGA-FITC staining
Figure S9. Labeling of the conidial surface of the I115S/I146S mutant with ConA-FITC or WGA-FITC. Note the absence of label in parental conidia.

## Slide 10
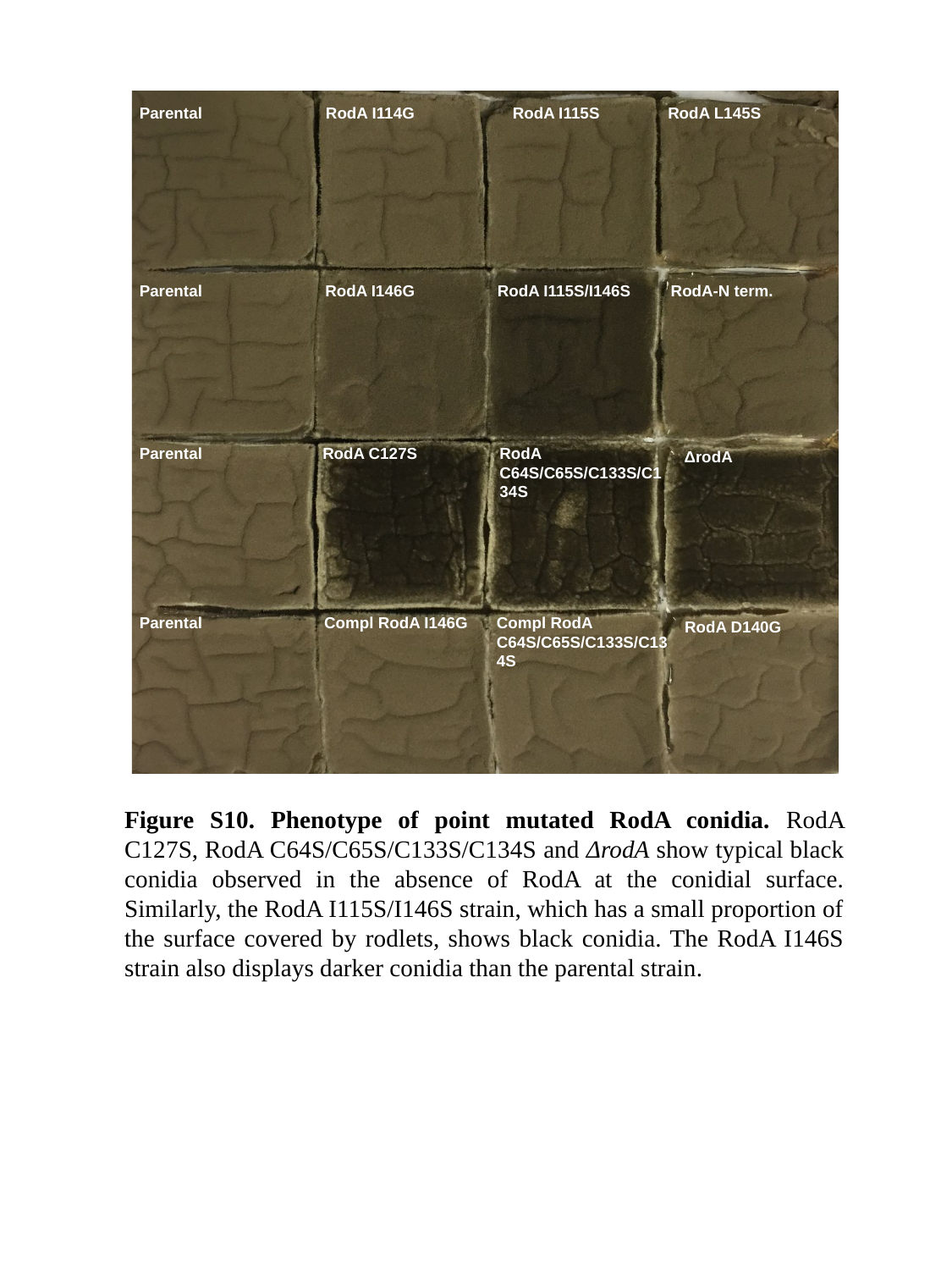

Parental
RodA I114G
RodA I115S
RodA L145S
Parental
RodA I146G
RodA I115S/I146S
RodA-N term.
Parental
RodA C127S
RodA C64S/C65S/C133S/C134S
ΔrodA
Parental
Compl RodA I146G
Compl RodA C64S/C65S/C133S/C134S
RodA D140G
Figure S10. Phenotype of point mutated RodA conidia. RodA C127S, RodA C64S/C65S/C133S/C134S and ΔrodA show typical black conidia observed in the absence of RodA at the conidial surface. Similarly, the RodA I115S/I146S strain, which has a small proportion of the surface covered by rodlets, shows black conidia. The RodA I146S strain also displays darker conidia than the parental strain.

## Slide 11
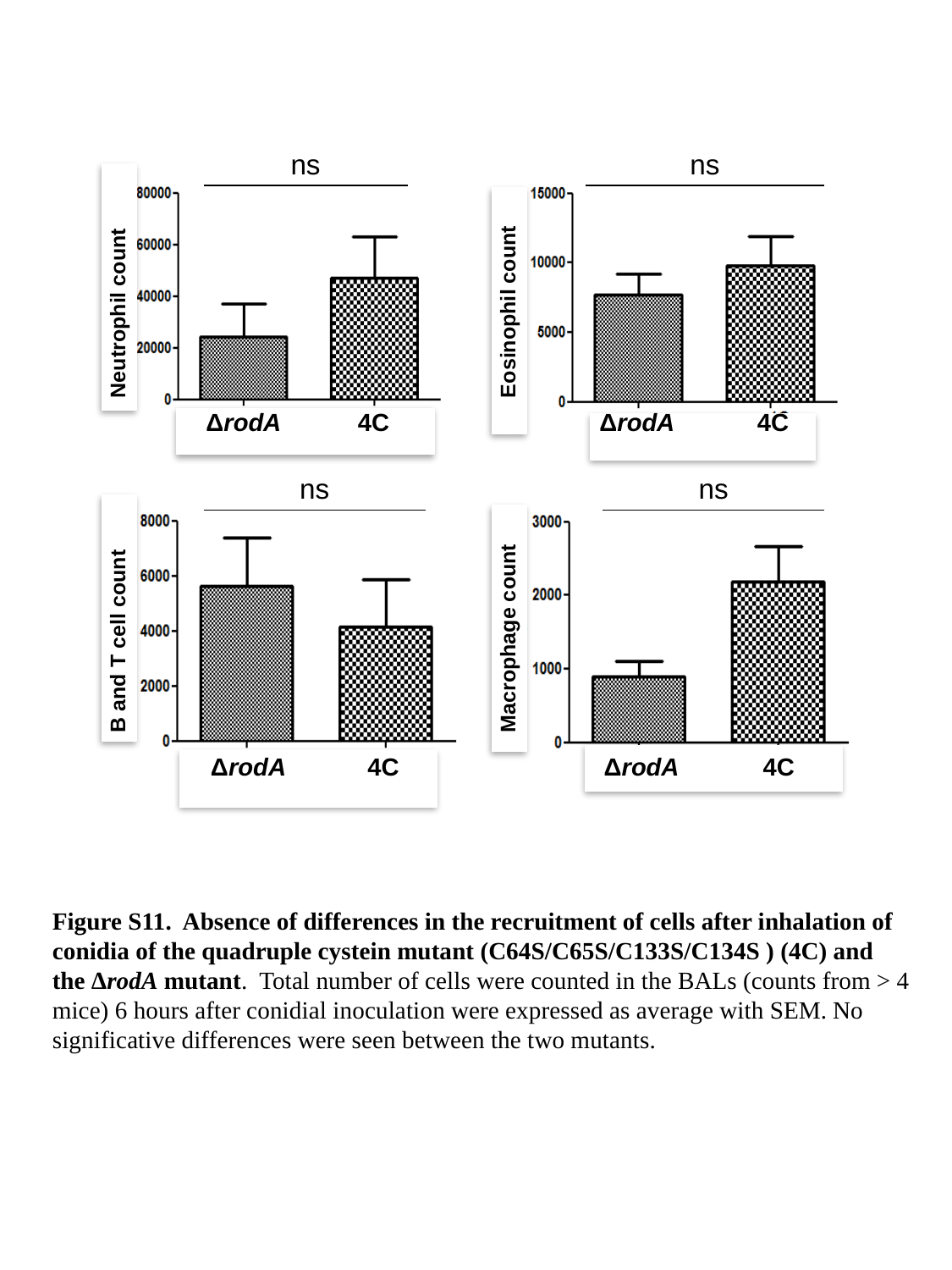

ns
ns
Neutrophil count
Eosinophil count
ΔrodA
4C
ΔrodA
4C
ns
ns
B and T cell count
Macrophage count
ΔrodA
4C
ΔrodA
4C
Figure S11. Absence of differences in the recruitment of cells after inhalation of conidia of the quadruple cystein mutant (C64S/C65S/C133S/C134S ) (4C) and the ∆rodA mutant. Total number of cells were counted in the BALs (counts from > 4 mice) 6 hours after conidial inoculation were expressed as average with SEM. No significative differences were seen between the two mutants.

## Slide 12
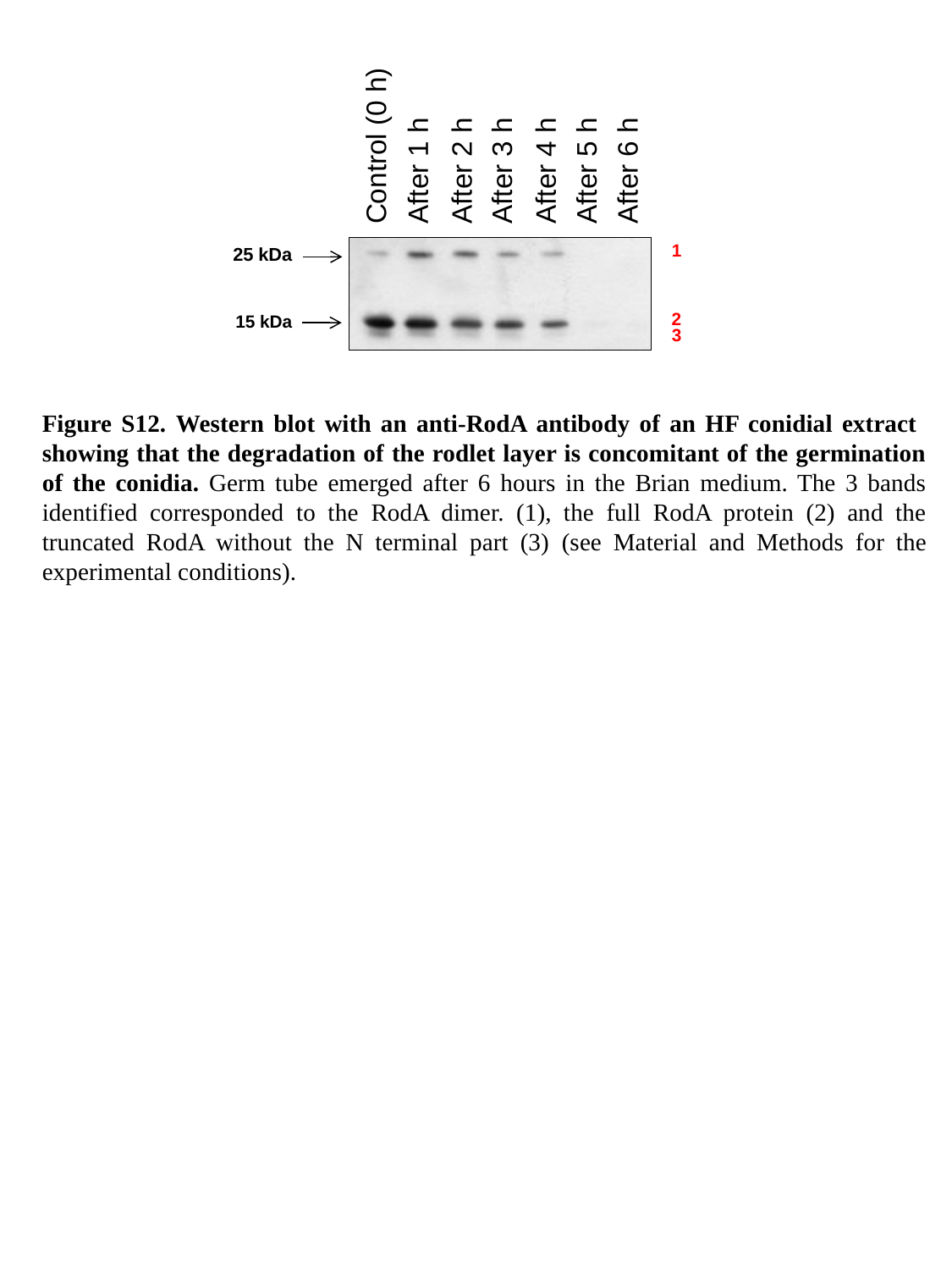

Control (0 h)
After 1 h
After 2 h
After 3 h
After 5 h
After 4 h
After 6 h
1
25 kDa
2
15 kDa
3
Figure S12. Western blot with an anti-RodA antibody of an HF conidial extract showing that the degradation of the rodlet layer is concomitant of the germination of the conidia. Germ tube emerged after 6 hours in the Brian medium. The 3 bands identified corresponded to the RodA dimer. (1), the full RodA protein (2) and the truncated RodA without the N terminal part (3) (see Material and Methods for the experimental conditions).
