# Supplementary material for: Assembly and disassembly of Aspergillus fumigatus conidial rodlets
Source: Cell Surf. 2019 Mar 6;5:100023. doi: 10.1016/j.tcsw.2019.100023 (PMC7389560; doi:10.1016/j.tcsw.2019.100023)
Supplement: Supplementary Table 1 [file mmc2.docx]

**Supplementary Table 1.** Statistics for the ensemble of 10 structures calculated for RodA (19-159)

Constraints (residues 19-159) ^a^ Energies (kcal/mol)

Unambiguous restraints 1590 Total -5896 ± 32

Ambiguous distance restraints 987 Van der Waals -494 ± 26

Total number of distance restraints ^a^ 2577 Electrostatic -5312 ± 56

Intra-residue | j-i | = 0 942 Mean of pairwise RMSD (Å) (39-159) ^d^

Sequential | j-i | = 1 695 Backbone atoms N, CA, C', O 0.68 ± 0.18

Medium range 2 ≤ | j-i | ≤ 4 387 Heavy atoms 0.80 ± 0.19

Long range | j-i | > 4 535 Ensemble Ramachandran plot (39-159) ^b^

Backbone dihedral ϕ angle restraints ^b^ 80 Residues in most favoured regions 89.6%

Backbone dihedral ψ angle restraints ^b^ 80 additionally allowed 9.9%

Total Backbone dihedral angle restraints ^b^ 160 generously allowed 0.3%

disallowed 0.2%

Residual distance constraint violations ^c^ Structure Z scores (39-159) ^d^

Number ≥ 0.5 Å 8.0 ± 4 Second generation packing quality -2.25 ± 0.18

Number ≥ 0.3 Å 12 ± 4 Ramachandran plot appearance -1.18 ± 0.36

Number ≥ 0.1 Å 23 ± 5 Chi1/Chi2 rotamer normality -1.33 ± 0.36

RMS deviation from nOes (Å) 0.065 ± 0.019 Backbone conformation -9.53 ± 2.09

Residual dihedral angle constraint violations Unsatisfied H-bond donors per molecule ^d^ 10.5

Number ≥ 5.0 ° 1.6 ± 1.4 Unsatisfied H-bond acceptors per molecule ^d^ 1.3

RMS deviation from dihedrals (°) 0.96 ± 0.21 Bumps (39-159) ^d^ 6.5

^a^ Distance constraints used for structure calculations, which excluded fixed intra-residue distances. Statistics include the N-terminal Ser residue that is not part of *A. fumigatus* RodA.

^b^ From TALOS-N (Shen *et al.* 2013).

^c^ Structures were calculated using the log-normal potential in ARIA, which results in a higher number of violations than flat bottom potentials.

^d^ Values for the structured region (between residues 39 and 159).
