# Supplementary material for: Assembly and disassembly of Aspergillus fumigatus conidial rodlets
Source: Cell Surf. 2019 Mar 6;5:100023. doi: 10.1016/j.tcsw.2019.100023 (PMC7389560; doi:10.1016/j.tcsw.2019.100023)
Supplement: Supplementary Table 3 [file mmc4.docx]

| N° | Primer sequence | Name |
| --- | --- | --- |
| RodA (AFUA_5G09580, AFUB_057130) | | |
| 1 | 5’ ATTCGAGCTCGGTACGCATGCACTGGAGTATGGCTTACGGC 3’ | pUC19 SpHI RodA upstream region F |
| 2 | 5’ CCTATAGGACCTGAGTGATGCTTACAGGATAGAACCAAGGGC 3’ | RodA stop HPH^R^-βrec R |
| 3 | 5’ AATATGGTCCATCTAGTGCGCTACTCGTCGCTTCTGCTGTT 3’ | HPH^R^-βrec RodA downstream region F |
| 4 | 5’ CCAAGCTTGCATGCCGCATGCGGGGGTCTACGATCTTTTCT 3’ | RodA downstream region SpHI pUC19 R |
| 5 | 5’ CTGGTCAACCAGAAG**AGC**AAGCAGAACATT 3’ | RodA C127S  mutagenesis F |
| 6-7 | 5’ GCTCAGCTCTCT**TCCTCC**AACAAGGCCACC 3’ | RodA  C64S-C65S-C133S-C134S  Mutagenesis F |
|  | 5’ AAGCAGAACATTGCC**TCCTCC**CAGAACTCTCCT 3’ |  |
| 8 | 5’ TCCCC**GGC**ATTGGTATTCC 3’ | RodA I114G  Mutagenesis F |
| 9 | 5’ TCCCCATC**AGT**GGTATTCCAATCCAGG 3’ | RodA I115S  Mutagenesis F |
| 10 | 5’ AGCGGTTCC**AGC**ATTGGACTGGGTC 3’ | RodA L145S  Mutagenesis F |
| 11 | 5’ AGCGGTTCCCTC**GGA**GGACTGGGTCTT 3’ | RodA I146G  Mutagenesis F |
| 12 | 5’ AACTCTCCTTCC**GGT**GCCAGCGGTTC 3’ | RodA D140G  Mutagenesis F |
| 13 | 5’ TCACGGTGATGTCGTCAGGAACGGGGAAGCGAAGGGCGGCCACGGAGACGGCGAAAGCG 3’ | RodA signal peptide  WO N-terminal R |
| 14 | 5’ CGCTTTCGCCGTCTCCGTGGCCGCCCTTCGCTTCCCCGTTCCTGACGACATCACCGTGA3’ | RodA signal peptide WO  N-terminal F |

**Supplementary Table 3** Primers used for the construction of point mutated *rodA A. fumigatus* mutants

Legend Not underlined= pUC19 or HPH^R^-βrec DNA sequences; underlined= *A. fumigatus* DNA sequences; double underlined= Partial or total restriction enzyme sequences

Bold = RodA point mutations

F = forward primer; R = reverse primer
